# Novel Immunomodulatory Flagellin-Like Protein FlaC in *Campylobacter jejuni* and Other *Campylobacterales*

**DOI:** 10.1128/mSphere.00028-15

**Published:** 2015-12-02

**Authors:** Eugenia Faber, Eugenia Gripp, Sven Maurischat, Bernd Kaspers, Karsten Tedin, Sarah Menz, Aleksandra Zuraw, Olivia Kershaw, Ines Yang, Silke Rautenschlein, Christine Josenhans

**Affiliations:** a Institute for Medical Microbiology and Hospital Epidemiology, Hannover Medical School, Hannover, Germany; b German Center for Infection Research (DZIF), Hannover, Germany; c Institute of Microbiology and Epizootics, Free University, Berlin, Germany; d Institute of Animal Physiology, Ludwig Maximilian University Munich, Munich, Germany; e Institute of Veterinary Pathology, Free University Berlin, Berlin, Germany; f Clinic for Poultry, Veterinary Medical School, Hannover, Germany; Swiss Federal Institute of Technology Lausanne (EPFL)

**Keywords:** *Campylobacter*, host-pathogen interaction, immune response, TLR5, flagellin

## Abstract

The human diarrheal pathogens *Campylobacter jejuni* and *Campylobacter coli* interfere with host innate immune signaling by different means, and their flagellins, FlaA and FlaB, have a low intrinsic property to activate the innate immune receptor Toll-like receptor 5 (TLR5). We have investigated here the hypothesis that the unusual secreted, flagellin-like molecule FlaC present in *C. jejuni*, *C. coli*, and other *Campylobacterales* might activate cells via TLR5 and interact with TLR5. FlaC shows striking sequence identity in its D1 domains to TLR5-activating flagellins of other bacteria, such as *Salmonella*, but not to nonstimulating *Campylobacter* flagellins. We overexpressed and purified FlaC and tested its immunostimulatory properties on cells of human and chicken origin. Treatment of cells with highly purified FlaC resulted in p38 activation. FlaC directly interacted with TLR5. Preincubation with FlaC decreased the responsiveness of chicken and human macrophage-like cells toward the bacterial TLR4 agonist lipopolysaccharide (LPS), suggesting that FlaC mediates cross-tolerance. *C. jejuni flaC* mutants induced an increase of cell responses in comparison to those of the wild type, which was suppressed by genetic complementation. Supplementing excess purified FlaC likewise reduced the cellular response to *C. jejuni*. *In vivo*, the administration of ultrapure FlaC led to a decrease in cecal interleukin 1β (IL-1β) expression and a significant change of the cecal microbiota in chickens. We propose that *Campylobacter* spp. have evolved a novel type of secreted immunostimulatory flagellin-like effector in order to specifically modulate host responses, for example toward other pattern recognition receptor (PRR) ligands, such as LPS.

**IMPORTANCE** Flagellins not only are important for bacterial motility but are major bacterial proteins that can modulate host responses via Toll-like receptor 5 (TLR5) or other pattern recognition receptors. *Campylobacterales* colonizing the intestinal tracts of different host species harbor a gene coding for an unusual flagellin, FlaC, that is not involved in motility but is secreted and possesses a chimeric amino acid sequence composed of TLR5-activating and non-TLR5-activating flagellin sequences. *Campylobacter jejuni* FlaC activates cells to increase in cytokine expression in chicken and human cells, promotes cross-tolerance to TLR4 ligands, and alters chicken cecal microbiota. We propose that FlaC is a secreted effector flagellin that has specifically evolved to modulate the immune response in the intestinal tract in the presence of the resident microbiota and may contribute to bacterial persistence. The results also strengthen the role of the flagellar type III apparatus as a functional secretion system for bacterial effector proteins.

## Abstract (precis)

Flagellins not only are important for bacterial motility but are major bacterial proteins that can modulate host responses via Toll-like receptor 5 (TLR5) or other pattern recognition receptors. *Campylobacterales* colonizing the intestinal tracts of different host species harbor a gene coding for an unusual flagellin, FlaC, that is not involved in motility but is secreted and possesses a chimeric amino acid sequence composed of TLR5-activating and non-TLR5-activating flagellin sequences. *Campylobacter jejuni* FlaC activates cells to increase in cytokine expression in chicken and human cells, promotes cross-tolerance to TLR4 ligands, and alters chicken cecal microbiota. We propose that FlaC is a secreted effector flagellin that has specifically evolved to modulate the immune response in the intestinal tract in the presence of the resident microbiota and may contribute to bacterial persistence. The results also strengthen the role of the flagellar type III apparatus as a functional secretion system for bacterial effector proteins.

## INTRODUCTION

*Campylobacter jejuni* and *Campylobacter coli* are bacterial pathogens which colonize different hosts. While they generally cause acute, self-limiting intestinal disease, such as diarrhea, in humans, they persist chronically in several different animal species, including avian and mammalian hosts. The basis of the interaction with their hosts, the causality of virulence in humans, and the differential outcomes of the infection or colonization of different mammalian and avian hosts are incompletely understood.

*C. jejuni* is highly motile due to bipolar flagella, a characteristic which plays an important role in colonization of the host (1–3). Based on whole-genome information and functional characterization, the flagellum is predicted to have a conserved composition of basal body, hook, and filament portions. The flagellar filament is composed of two flagellins, FlaA and FlaB, which share high amino acid identity between each other (4). In addition, the flagellar type III secretion system (T3SS) of *C. jejuni* is also involved in the secretion of several nonflagellar proteins with roles in virulence (5). Several putative effector proteins, such as Cia proteins, FlaC, Cj0977, and FspA, are secreted through the flagellar T3SS of *C. jejuni* and influence the interaction with the host environment (5–8).

Canonical flagellin molecules, such as *Salmonella* FliC, are recognized by the cellular innate immune receptor, Toll-like receptor 5 (TLR5), of eukaryotic cells (9, 10). Two major conserved regions of flagellin, which also contribute to filament formation and motility, are responsible for TLR5 recognition (11, 12). Recently, Yoon et al. (12) crystallized the exodomain of TLR5 from zebrafish in a complex with FliC flagellin of *Salmonella enterica* serovar Typhimurium, known as a strong activating ligand of TLR5 (12). This study provided the first structural details for the interaction of TLR5 with flagellin. Two primary binding interfaces, A and B, within the folded D1 domains of the FliC antiparallel helices were defined to be involved in the specific binding of TLR5.

Interestingly, several studies demonstrated that flagellins of *Alpha*- and *Epsilonproteobacteria*, including *C. jejuni* and *Helicobacter pylori*, have a low intrinsic propensity to activate TLR5 (13–16), suggesting that these pathogens evolved an important mechanism to escape from the innate immune response of the host without compromising motility. The three-dimensional (3-D) structure of the flagellin multimer, the conformation of the alpha-helical flagellin D1 and D2 monomeric domains, and the surface-exposed epitopes in flagellin multimers are predicted to differ between the activating (*Salmonella*) and nonactivating (*Campylobacter*) flagellin variants (17). In general, TLR5 recognition of pathogenic and nonpathogenic bacteria seems to play a crucial role, in particular in the intestinal tract, as demonstrated by several studies using TLR5-deficient mice (18, 19; for a review, see reference 20). On one hand, TLR5 signaling is involved in the maintenance of immune homeostasis between the commensal microbiota and the host immune system (21), and on the other hand, it appears to play an important role in the defense against acute enterobacterial infection (19, 22, 23).

Due to the zoonotic potential of *C. jejuni* and its ability to colonize different hosts, the possible species-specific recognition of *C. jejuni*’s microbe-associated molecular patterns (MAMPs) by TLRs and other pattern recognition receptors (PRRs) should be considered while analyzing its interaction with different hosts. Several reports have shown the activation of NF-κB and mitogen-activated protein kinases (MAPKs) and the induction of different cytokines upon *C. jejuni* infection in both human and chicken cells, suggesting that *C. jejuni* is able to trigger innate immune responses in different hosts (16, 24–29). On the other hand, the outcomes of activation by *C. jejuni* vary between different host species. Indeed, species-specific properties of TLRs and their recognition have already been described. Human, chicken, and mouse TLR5 can recognize flagellin differentially (30, 31), and also chicken TLR4 appears to exhibit species-specific responses to bacterial lipopolysaccharide (LPS) or lipo-oligosaccharide (32).

The aim of the present work was to address the function of the unusual flagellin-like protein FlaC of *C. jejuni*. Until now, only one study has analyzed FlaC functionally, and it demonstrated that FlaC secretion depends on the flagellar apparatus (7). Additionally, the binding of FlaC to epithelial cells and its influence on cell invasion was previously demonstrated; however, no precise function could be attributed to this protein so far. Due to its high amino acid similarity with other flagellins, we hypothesized that FlaC may be able to interact with TLR5 and may thereby modulate host interaction and the innate immune response.

We report here that FlaC is conserved in various intestinal nonsheathed *Campylobacter* and *Helicobacter* species. Purified *C. jejuni* FlaC was tested for immunostimulatory properties on different cell types. FlaC activated cells, interacted with TLR5 *in vitro*, and influenced the responsiveness of chicken and human macrophage-like cells toward subsequent exposure to bacterial TLR4 ligands. We propose that *Campylobacter* spp. and related bacteria have evolved a novel type of secreted flagellin-like effector protein in order to modulate host responses and bacterial persistence.

## RESULTS

### *In silico* comparison of *C. jejuni* FlaC with other flagellin molecules—evidence for a chimeric amino acid sequence.

The gene *flaC* is conserved within the species *C. jejuni*. In order to identify potential FlaC orthologues in other *Campylobacter* species, we performed a BLAST search with *C. jejuni* FlaC. We found the protein to be conserved in eight other *Campylobacter* species (Table 1; see Fig. S1 and S2 in the supplemental material). An extended search for FlaC orthologues in all currently available genomes of different *Campylobacter* species revealed three aflagellated species, *C. gracilis*, *C. hominis*, and *C. ureolyticus*, where the FlaC open reading frame seems to be lacking. Interestingly, FlaC appears not to be restricted to the genus *Campylobacter*, since three intestinal *Helicobacter* species without flagellar sheaths and a commensal epsilonproteobacterium of the ruminant stomach, *Wolinella succinogenes*, possess clear FlaC orthologues, exhibiting 43% to 47% amino acid identity with *C. jejuni* FlaC (Table 1). Comparative analysis of the *flaC* gene context in different *flaC*-containing genomes revealed that genomes of more closely related *Campylobacter* species, e.g., *C. jejuni*, *C. coli*, and *C. fetus*, share the same *flaC* genetic neighborhood but that less closely related species seem to harbor *flaC* in a different genomic context (Fig. S1 and  S3). *flaC* gene sequences between different *C. jejuni* strains of different phylogenetic lineages showed ≤3.6% interstrain nucleotide polymorphisms (≥96.4% identical nucleotides) in both synonymous and nonsynonymous nucleotide sites (the ratio of the number of nonsynonymous substitutions to the number of synonymous substitutions [*K_a_/K_s_*] was ≤3.5) (data not shown).

10.1128/mSphere.00028-15.1 Figure S1  *flaC* and its genetic neighborhood in different *Campylobacter* species. Gene order and annotation, which are schematically depicted, were extracted from available whole-genome sequences in databases. Download Figure S1, PDF file, 0.1 MB. Copyright © 2015 Faber et al. 2016 Faber et al. This content is distributed under the terms of the Creative Commons Attribution 4.0 International license.

10.1128/mSphere.00028-15.2 Figure S2  (A) Unweighted pair group method using average linkages (UPGMA) tree of FlaC from different *Campylobacter* species, based on the protein sequence of FlaC, generated by MEGA 5.2; (B) maximum-likelihood tree (bootstrap method, 500 replicates) of FlaC proteins from different *Campylobacter* species, based on protein sequences of FlaC, generated by MEGA 5.2. *, *P* < 0.05. Download Figure S2, PDF file, 0.04 MB. Copyright © 2015 Faber et al. 2016 Faber et al. This content is distributed under the terms of the Creative Commons Attribution 4.0 International license.

10.1128/mSphere.00028-15.3 Figure S3  Gene organization upstream of *flaC* and cotranscript detection in *C. jejuni* 11168. Cj0721, 0722, and 0723 are tandemly positioned directly upstream of *flaC*, likely forming a gene cluster. To predict whether these genes are cotranscribed with *flaC*, PCRs were performed on cDNA of *C. jejuni* strain 11168 grown *in vitro*. DNA of the same strain served as a control for the efficiency of the PCR. We used several primer combinations which span the regions within each of the four genes and regions between the genes (shown in the graph; the locations of primers are indicated by colored arrows and PCR products by numbered connection lines between arrows). Download Figure S3, PDF file, 0.3 MB. Copyright © 2015 Faber et al. 2016 Faber et al. This content is distributed under the terms of the Creative Commons Attribution 4.0 International license.

**TABLE 1:** *C. jejuni* FlaC orthologues in *Campylobacter* spp. and other *Campylobacterales*^a^

Species and strain name (for genomic information)	Species host(s)/niche(s)	% amino acid identity to *C. jejuni* FlaC
*C. coli* RM2228	Cattle, chicken/intestinal tract	95
*Campylobacter upsaliensis* JV21	Cat, dog/intestinal tract	84
*Campylobacter lari* RM2100	Cattle, chicken, wild birds/intestinal tract	68
*Campylobacter showae* CSUSNWCD	Dog, human/intestinal tract	50
*Campylobacter rectus* RM3267	Dog/intestinal tract	49
*C. fetus* subsp*. fetus* 82.40	Diverse/intestinal tract, urogenital tract	49
*Campylobacter curvus* 525.92	Dog/intestinal tract	48
*H. pullorum* MIT 98-5489	Chicken, human/intestinal tract	47
*H. canadensis* MIT 98-5491	Wild birds, human/intestinal tract	45
*H. winghamensis* ATCC BAA 430	Human/intestinal tract	44
*W. succinogenes* DSM 1740	Cattle/intestinal tract	43
*C. concisus* 13826	Cat, dog, human/intestinal tract	39

a None of the species had flagellar sheaths.

**FIG 1:**
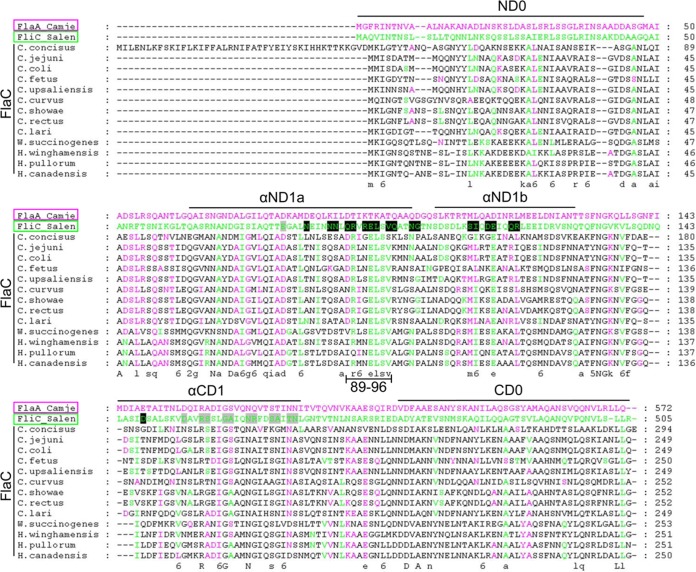
ClustalOmega alignment of FlaC protein sequences. *C. jejuni* (Camje) FlaA (pink, a representative of non-TLR5-stimulatory flagellins), *S. enterica* (Salen) FliC (green, a paradigm for a TLR5-activating flagellin), and FlaC protein sequences of various *Epsilonproteobacteria* were aligned with ClustalOmega (http://www.ebi.ac.uk/Tools/msa/clustalo) and visualized in GeneDoc (https://www.psc.edu/index.php/user-resources/software/genedoc). The locations of the D0 and D1 domains are indicated above the sequences. Residues of FliC involved in TLR5 binding and activation are shaded in gray (primary interface A) and in black (primary interface B) (according to reference 12). Residues of FlaC identical to those in *C. jejuni* FlaA or *S. enterica* FliC are colored accordingly in pink or green, respectively. A consensus score is shown underneath the alignment. Only flagellin sequence domains D0 and D1 present in FlaC are depicted; since the D2 and D3 domains are largely absent from FlaC orthologues, these domains have been omitted from the alignment.

We generated an amino acid alignment of all currently identified FlaC orthologues with *Salmonella enterica* FliC, representing canonical TLR5-activating flagellin, and *Campylobacter* FlaA, representing non-TLR5-activating flagellin (13–16) (Fig. 1). In comparison to other bacterial flagellins, *Campylobacter* FlaC shows a striking amino acid sequence similarity in crucial residues of its D1 domains to TLR5-stimulating flagellins of other bacteria, such as *Salmonella*. In particular, the D1 region comprising amino acids 89 to 96 of FliC within its primary interface B (12), which is important for TLR5 binding and activation (11, 12), appears to be conserved in *Campylobacter* FlaC (Fig. 1). On the other hand, some other amino acids within the FlaC D1 domains are more similar to the TLR5-nonstimulating domains that were previously described for the *Campylobacterales Campylobacter* and *Helicobacter* (13–16, 33), suggesting a chimeric amino acid sequence of FlaC between TLR5-stimulating and non-TLR5-stimulating flagellins (Fig. 1).

### Characterization of *C. jejuni flaC* mutants and bacterial subcellular localization of FlaC in *C. jejuni* 11168 and 81-176.

**FIG 2:**
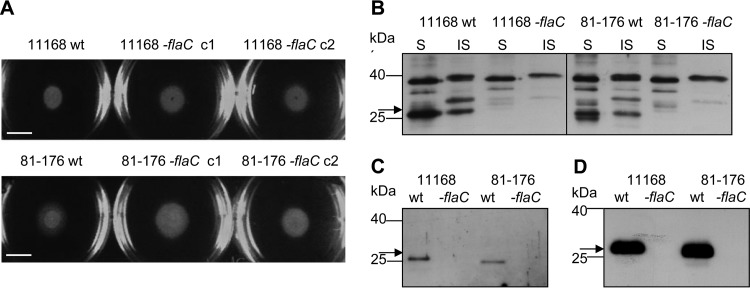
Characterization of *C. jejuni flaC* mutants and subcellular localization of FlaC. (A) Motility of the *C. jejuni* 11168 and 81-176 wild types (wt) and two corresponding *flaC* mutants (clone 1 and 2 [c1 and c2, respectively]) of each strain. A representative soft-agar motility plate from at least three independent assays shows the motility areas of all bacterial strains (swim diameter of ca. 10 mm) after 2 days of incubation at 37°C (scale bar, 10 mm). Corresponding motility-negative controls of *C. jejuni* (*flgR* mutant) always exhibited a swim halo diameter of <1 mm under the same conditions, while *flaC*-complemented bacteria reproducibly had a swim diameter similar to that of the wild type and *flaC* mutant (not shown). (B to D) Subcellular localization of FlaC in *C. jejuni* bacterial fractions. Comparative Western blot analyses of whole-cell lysates and different fractions of *C. jejuni* 11168 and 81-176 wild-type and *flaC*-mutants grown under microaerobic conditions were performed using polyclonal rabbit FlaC antiserum (dilution, 1:5,000). (B) S, soluble bacterial fraction; IS, insoluble bacterial fraction. (C) Surface/flagellar proteins. (D) Secreted proteins. Expression of FlaC was also investigated under anaerobic conditions. Western blot analyses of whole-cell lysates and different fractions of wild-type and *fl*aC mutants grown under anaerobic conditions yielded comparable results (not shown). Our estimate from comparative Western blots was that approximately 2 µg of FlaC was secreted per 10^9^ bacteria, and 100 to 200 ng of cell-bound FlaC was present in the same number of bacteria.

In order to characterize the function and localization of FlaC in the bacterial context, we generated *C. jejuni* mutants lacking FlaC in different strains by allelic-exchange mutagenesis. These mutants of strains 11168 and 81-176 were first analyzed with regard to their motility in comparison to that of their respective wild-type bacteria in motility agar plates (Fig. 2A). All bacteria showed comparable levels of positive motility after 2 days of incubation, indicating that the absence of FlaC has no perceptible impact on bacterial motility. Swimming assays in liquid or assays for biofilm formation did not reveal differences either (data not shown). These findings are in agreement with results previously published by Song et al. for a nonrelated *C. jejuni* strain, TGH9011 (7).

Western blot analyses of whole bacterial lysates and different fractions of *C. jejuni* wild-type and *fla*C mutants using anti-FlaC antiserum (see Materials and Methods and Fig. 2B) revealed that FlaC has a molecular mass of about 28 kDa, with minor mass variation between native FlaCs of the two strains 11168 and 81-176. FlaC was present in the soluble and the insoluble fractions of the bacterial lysates, and a small FlaC fraction could be detected in the surface-associated protein fractions. A large proportion of the protein (>90%) was identified in the secreted protein fraction, corroborating previously published results (7).

### Antibody responses to *C. jejuni* FlaC in infected chickens suggest its expression *in vivo*.

**FIG 3:**
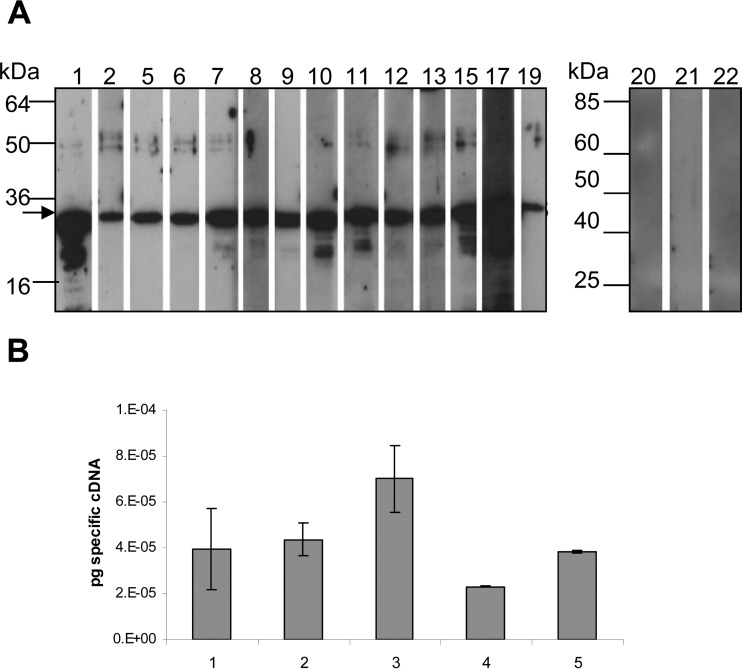
Immune response in infected chickens to *C. jejuni* FlaC. (A) Adaptive immune response to recombinant *C. jejuni* FlaC in *Campylobacter*-infected chickens. Western blot analyses were performed to investigate and compare the reactivities of whole sera from *Campylobacter*-infected (lanes 1 to 19) and noninfected (lanes 20 to 22) chickens. Chicken sera were used at a dilution of 1:10,000 and detected with horseradish-peroxidase-coupled anti-chicken antibody. (B) *In vivo* expression of *flaC* in chicken cecal tissue. Transcript levels of *flaC* were determined by using quantitative RT-PCR of total RNA extracted from the ceca of five chickens which had been experimentally infected with *C. jejuni* strain RB922 (48). The amounts of specific *flaC* cDNA (in picograms) were normalized to *C. jejuni* 16S rRNA gene amounts determined in each animal.

Samples of 22 chickens which differed in age, breed, and husbandry conditions (see Table S1 in the supplemental material) were analyzed for an infection with *Campylobacter*. We plated cloacal swabs on blood agar and screened the recovered bacteria visually and microscopically for their morphology. Furthermore, we clone-purified potential *Campylobacter* colonies from the swabs and analyzed them using PCR with universal *Campylobacter* primers (not shown), confirming *Campylobacter* identity. Sera of 14 *Campylobacter*-infected chickens were subsequently tested in a Western blot analysis against purified recombinant FlaC. Although the intensities of the signals varied depending on the individual bird, each serum from a *Campylobacter*-positive chicken was reactive against recombinant purified FlaC of *C. jejuni*, even at a high serum dilution of 1:10,000 (Fig. 3A). In contrast, sera from *Campylobacter*-free chickens (specific-pathogen-free [SPF] animals 20 to 22) showed no reactivity to FlaC (Fig. 3A). These results may support the possibility that FlaC is expressed *in vivo* in chicken. In order to verify directly whether FlaC is expressed *in vivo* in the chicken cecum, quantitative real-time PCR was performed on cecal tissues from experimentally infected chickens. The *flaC* transcript was indeed present in chicken cecal tissue (Fig. 3B). The FlaC-recognizing chicken sera also detected the *C. jejuni* major flagellin, FlaA, in bacterial fractions (not shown).

10.1128/mSphere.00028-15.4 Table S1  Origin of sera from *Campylobacter*-positive and -negative chickens. Download Table S1, PDF file, 0.01 MB. Copyright © 2015 Faber et al. 2016 Faber et al. This content is distributed under the terms of the Creative Commons Attribution 4.0 International license.

### Purified *C. jejuni* FlaC activates human and chicken cells.

For further functional characterization, *C. jejuni* FlaC was expressed in *Escherichia coli* and purified under denaturing conditions using metal affinity chromatography (13). The purified protein was then dialyzed several times and gel eluted to render it highly pure and free from contamination with LPS, as previously established (see Materials and Methods and reference 13). For the investigation of the proposed host cell stimulatory properties of FlaC, we coincubated highly purified *C. jejuni* FlaC with human and chicken macrophage-like and epithelial cell lines and determined cytokine release into the supernatants. Flagellins acting via TLR5 have been shown to induce interleukin 8 (IL-8) secretion in human cells (34). Unexpectedly, enzyme-linked immunosorbent assay (ELISA) measurements using supernatants of human HEK293T, THP-1, and colon intestinal epithelial Lovo cells did not reveal significantly increased secreted IL-8 or IL-1β levels after 4 to 5 h of coincubation with highly pure FlaC (not shown), although these cells secreted cytokines in response to *Salmonella* FliC used as a control.

**FIG 4:**
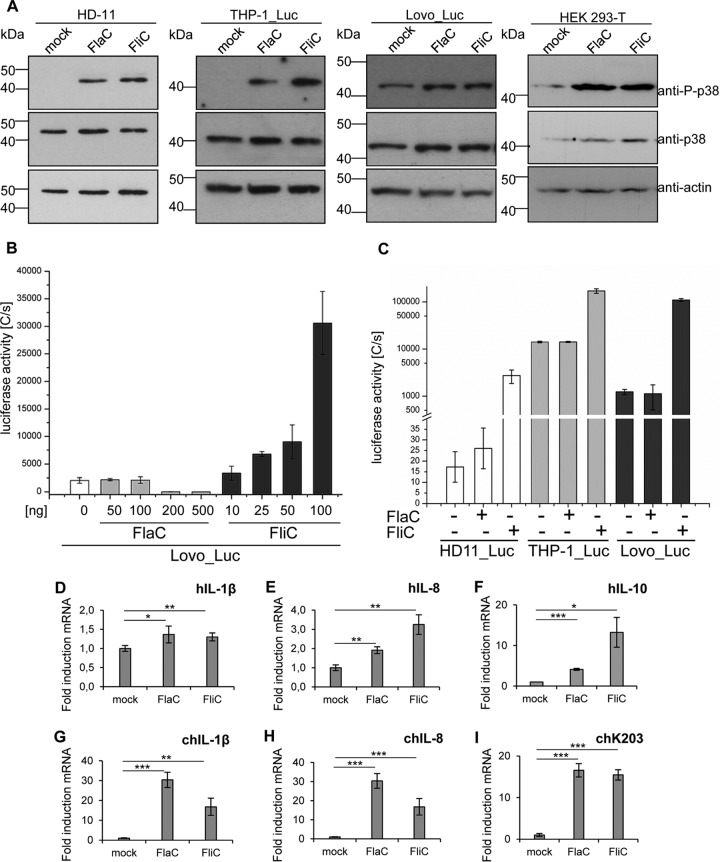
Effect of purified *C. jejuni* FlaC on human and chicken cell signal transduction. (A) p38 MAP kinase phosphorylation in human Lovo_Luc, THP-1_Luc, HEK293T, and chicken HD-11 cells was analyzed by Western immunoblotting after coincubation of the cell lines with ultrapure recombinant *Salmonella* FliC (100 ng) or ultrapure recombinant *C. jejuni* FlaC (200 ng) for 4 h (see Materials and Methods). Densitometry of P-p38 band intensities was performed, and results were normalized against those for the respective p38 signal and the actin loading control band in each lane. Normalized intensity values indicated that for all 3 cell lines, P-p38 was at least 5-fold enhanced by FlaC over levels in the mock-incubated control. (B) Analysis of the concentration-dependent response of Lovo cells, stably transfected with an NF-κB luciferase reporter gene, toward *Salmonella* FliC (positive control) or recombinant *C. jejuni* FlaC. (C) HD-11, THP-1, and Lovo cells stably transfected with an NF-κB luciferase reporter were coincubated with *Salmonella* FliC (25 ng) or recombinant *C. jejuni* FlaC (100 ng) for 3 h in 96-well plates and analyzed for NF-κB-driven luciferase expression using the SteadyGlo luciferase assay. The luciferase activities in panels B and C are given in luminescence values as photon counts per second (C/s). (D to I) Quantitative RT-PCR of cytokine mRNA induction by *C. jejuni* FlaC in human and chicken cells. Levels of induction of hIL-1β (D), hIL-8 (E), and hIL-10 (F) by *C. jejuni* FlaC and *S*. Typhimurium FliC in human macrophages (THP-1) and of chIL-1β (G), chIL-8 (H), and chK203 (I) in chicken macrophages (HD-11) are shown. Both cell types were stimulated for 2 h with recombinant ultrapure proteins FlaC (500 ng) and FliC (300 ng). Isolated RNA was analyzed by quantitative RT-PCR. Transcript values were normalized to human or chicken GAPDH values and are presented as fold increases of mRNA levels compared to the level in a mock-coincubated control. Mean values and standard deviations from triplicate measurements are shown. Significant *P* values are indicated by asterisks (Student’s *t* test, unpaired, one-sided) as follows: *, 0.01 ≤ *P* ≤ 0.05; **, 0.001 ≤ *P* ≤ 0.01; and ***, *P* ≤ 0.001.

Since flagellins can activate several different and mutually overlapping signaling pathways (35–37), e.g., those mediated by TLR5 and inflammasomes, we performed further activation analyses in order to test stimulating properties of *C. jejuni* FlaC downstream of TLRs, at the level of transcription factor NF-κB and mitogen-activated protein kinases (MAPKs). MAPK p38 and extracellular signal-regulated kinase (ERK) have been described to be strongly activated by flagellins, e.g., the canonical TLR5 ligand *Salmonella enterica* FliC (34, 38). To investigate whether *C. jejuni* FlaC is able to activate the MAPK cascade, we coincubated human and chicken cells with highly purified FlaC and analyzed p38 and ERK phosphorylation in Western blots. Highly pure FlaC led to an enhanced phosphorylation of p38 (Fig. 4A) and ERK (data not shown) in human and chicken cells at different time points of coincubation (2 h, 4 h), as did the control protein FliC. For the analysis of NF-κB activation, human THP-1, Lovo, and chicken HD-11 cells stably transfected with an NF-κB-dependent luciferase reporter construct were incubated with highly pure recombinant *C. jejuni* FlaC or with highly pure recombinant *Salmonella* FliC as a positive control (Fig. 4B and C). The results were quantitated using luciferase measurements. Chicken HD-11 and human THP-1 cells showed significantly enhanced NF-κB activation levels after coincubation with purified FliC (Fig. 4C). FlaC, like the mock control, did not significantly enhance NF-kB-dependent luciferase in human or chicken cells (Fig. 4B and C), whereas *Salmonella* FliC activated cells in a concentration-dependent manner, as expected (shown for Lovo cells in Fig. 4B).

A stimulated TLR5 signaling cascade by bacterial flagellin leads to the expression of numerous proinflammatory cytokines (35). Since no significantly elevated IL-8 secretion could be identified after exposure of different human cell types (THP-1, Lovo, HEK293 cells) (data not shown) to purified FlaC, we assayed TLR5 downstream signaling with regard to transcriptional activation of cytokine genes. For this purpose, we coincubated human (THP-1) and chicken (HD-11) macrophage-like cells with purified FlaC or with FliC as a positive control for 2 h. Quantitative analysis via real-time PCR revealed significantly enhanced transcript amounts of human IL-1β (hIL-1β) (Fig. 4D), hIL-8 (Fig. 4E), and hIL-10 (Fig. 4F) in the human cells. In chicken cells, we determined even >10-fold-increased transcript levels of chicken IL-1β (chIL-1β) (Fig. 4G), chIL-8 (Fig. 4H), and the chicken monocyte chemotactic cytokine chK203 (Fig. 4I) (39) after a 2-h exposure to purified FlaC, similar to what occurred with the positive control, FliC. As an additional control, to verify TLR5-dependent cell activation by FlaC, we used HEK293 cells transiently transfected with human TLR5 (hTLR5) expression plasmid in accordance with previously published controls (33). The transiently transfected hTLR5 cells showed a slight but significant induction of IL-8 secretion when coincubated with FlaC (Fig. S4), while a strong, significant induction was demonstrated with *Salmonella* FliC. However, when cytokine mRNAs were quantitated in the hTLR5-transfected cells, we again noted a significant induction of IL-10 mRNA by both FlaC (6-fold) and FliC (4-fold) in comparison to levels of induction in the hTLR5 control (data not shown).

10.1128/mSphere.00028-15.5 Figure S4  IL-8 secretion of TLR5 plasmid-transfected HEK293T cells coincubated with FlaC and FliC. HEK293T cells were mock transfected or transiently transfected for human TLR5 (hTLR5) and 48 h after transfection coincubated with recombinant highly purified FlaC (200 ng) or *S*. Typhimurium FliC (100 ng, positive control) for 5 h. IL-8 secretion was determined using a human IL-8 ELISA (BD OptEIA). Mean values and standard deviations from duplicate biological experiments and triplicate measurements for each experiment are shown. FlaC led to a slight induction of IL-8 secretion in the TLR5 plasmid-transfected cells. Ultrapure *E. coli* LPS did not lead to induction of IL-8 secretion in this setting (data not shown). Significance of differences was calculated by Student’s *t* test (unpaired, one-sided), as follows: ***, ≤0.001; **, ≤0.01; *, ≤0.05; n.s., not significant. Download Figure S4, PDF file, 0.03 MB. Copyright © 2015 Faber et al. 2016 Faber et al. This content is distributed under the terms of the Creative Commons Attribution 4.0 International license.

### Recombinant purified FlaC is able to bind to cells and to physically associate with TLR5.

**FIG 5:**
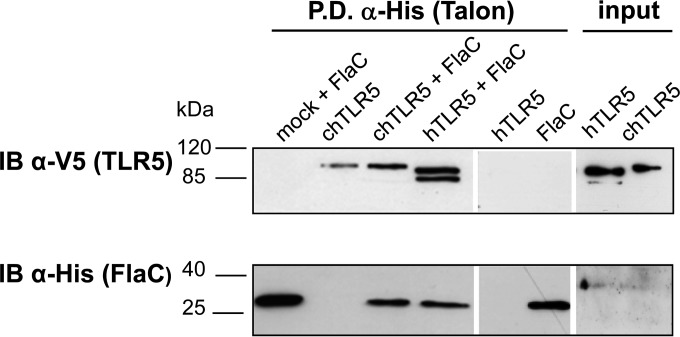
Interaction of *C. jejuni* FlaC with TLR5 *in vitro*. Binding of *C. jejuni* FlaC to TLR5 was tested by pulldown assay. Cleared cell lysates from TLR5 plasmid-transfected (hTLR5, human; chTLR5, chicken; both expressed as a V5-tagged protein fusion) or empty-plasmid-transfected cells were generated and coincubated with purified *C. jejuni* 6×His-FlaC. Pulldown against the 6×His tag fused to FlaC was performed using Talon Co^2+^ resin. Protein detection on Western blots was performed using rabbit anti-6×His antiserum (diluted 1:1,000; Rockland) for FlaC, and anti-V5 antibody (mouse monoclonal, 1:5,000; Invitrogen) for the detection of hTLR5 and chTLR5 (V5 tagged). The input was cleared cell lysates of TLR5-V5 plasmid-transfected HEK293T cells that were used for the pulldown. IB, immunoblotting; P.D., pulldown.

In order to test whether FlaC can bind to cells, we added gel-extracted highly pure recombinant *C. jejuni* FlaC (see Materials and Methods) to live chicken HD-11 or human HEK293T cells. Cells were coincubated with FlaC for 1 h, washed extensively, lysed, and fractionated. The lysate fractions (soluble and insoluble) were analyzed on SDS gels for FlaC binding. We detected FlaC binding both to human and to chicken cells, predominantly in the insoluble cell fractions containing membrane constituents (see Fig. S5 in the supplemental material). Binding of the positive-control protein *Salmonella* FliC was also demonstrated with both cell lines (Fig. S5A and B). While full-length FliC was recovered in HEK293 cell fractions (Fig. S5A), in the case of chicken macrophage-like HD-11 cells, only a FliC band of lower molecular mass, probably representing a processed form of FliC, could be identified (Fig. S5B). Subsequently, we tested for a proposed direct physical interaction of FlaC with TLR5 using a pulldown approach against the 6×His tag of recombinant FlaC. For this purpose, we used precleared lysates of HEK293T cells that had been transiently transfected with TLR5 expression plasmid, expressing chicken or human TLR5 (11, 13), or with the corresponding empty vector. These lysates were incubated to interact with purified, 6×His-tagged FlaC. We noted a direct *in vitro* interaction of FlaC with hTLR5 in these assays (Fig. 5). chTR5 was also pulled down with FlaC; however, the chTLR5 control also slightly bound to the affinity beads in the absence of FlaC. Modeling the structure of the TLR5-FlaC heterotetramer according to the recently published TLR5 ectodomain crystal structure (12) using HexDock (HexServer) (40) proposed a thermodynamically favorable interaction interface between the two proteins, specifically at the FlaC D1 domain (Fig. S6). The modeled TLR5-FlaC heterotetramer arrangement has similar aspects but is not identical to the FliC-D1-TLR5 interface in the published crystal structure (12).

10.1128/mSphere.00028-15.6 Figure S5  Binding of *C. jejuni* FlaC to human and chicken cells. Human (HEK293T) (A) and chicken (HD-11) (B) cells were coincubated with 1 µg FlaC or 1 µg *Salmonella* FliC (positive control) for 1 h (both proteins as 6×His-tagged fusion proteins). After intensive washes, cell lysates were generated, fractionated, and analyzed on SDS gels. Bound FlaC was detected in the insoluble cell fractions using anti-FlaC polyclonal antiserum. Bound FliC was detected in the insoluble cell fractions using anti-6×His antibody. Enrichment of a low-molecular-mass band of ca. 35 kDa in the HD-11 chicken macrophage-like cell fraction (insoluble) coincubated with *Salmonella* FliC is noted. IB, immunoblot. Download Figure S5, PDF file, 0.3 MB. Copyright © 2015 Faber et al. 2016 Faber et al. This content is distributed under the terms of the Creative Commons Attribution 4.0 International license.

10.1128/mSphere.00028-15.7 Figure S6  Molecular docking of modeled *C. jejuni* FlaC with TLR5. The FlaC structure was modeled according to the flagellin FliC of *S*. Typhimurium (1ucuA; complete atomic model of the bacterial flagellar filament by electron cryomicroscopy [K. Yonekura, S. Maki-Yonekura, and K. Namba, Nature **424:**643–650, 2003]) using the Swiss model [N. Guex, M. C. Peitsch, and T. Schwede, Electrophoresis **30**(S1)**:**162–173, 2009]. Two FlaC molecules (blue) were docked to a dimer of the zebrafish TLR5 ectodomain (yellow; extracted from PDB accession number 3V47 [S. Yoon et al., Science **335:**859–864, 2012]) using HexDock (HexServer, a Fourier transform [FFT]-based protein docking server powered by graphics processors [G. Macindoe, et al., Nucleic Acids Research **38:**W445–W449, 2010]). The following default docking settings were used: docking_correlation 0, docking_refine 0, docking_grid_size 0.6, max_docking_solutions 3000, receptor_range_angle 180, docking_receptor_stepsize 7.5, ligand_range_angle 180, docking_ligand_stepsize 7.5, docking_r12_range 40, docking_r12_substeps 0, docking_main_scan 20, docking_main_search 25, docking_fft_device 1, and docking_fft_type 1. Download Figure S6, PDF file, 0.4 MB. Copyright © 2015 Faber et al. 2016 Faber et al. This content is distributed under the terms of the Creative Commons Attribution 4.0 International license.

### *C. jejuni* FlaC antagonizes the activating effect of the TLR4 ligand bacterial lipopolysaccharide.

**FIG 6:**
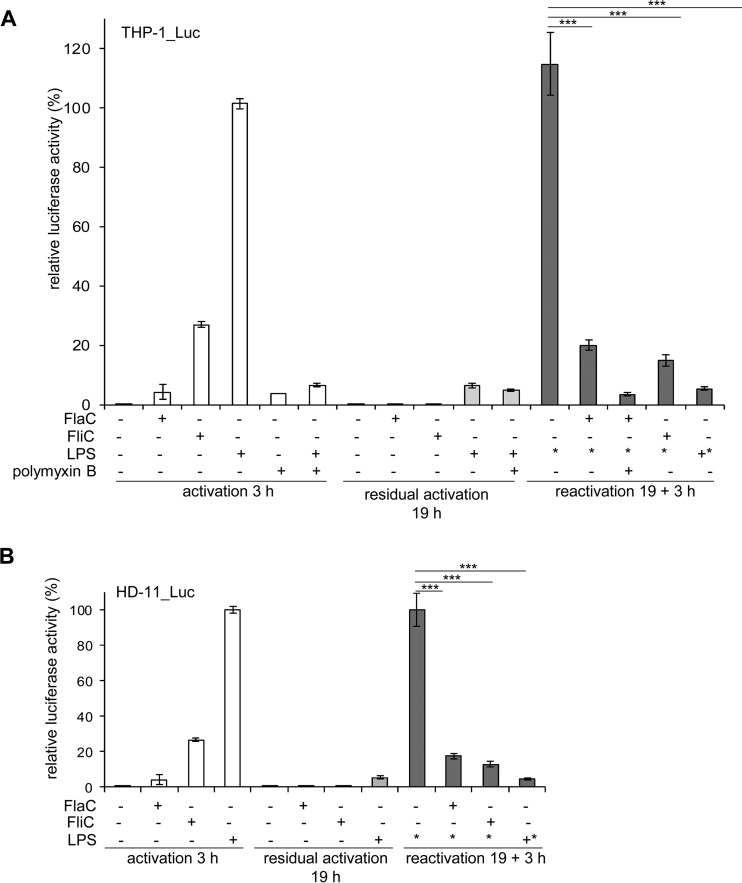
*C. jejuni* FlaC antagonizes the activating effect of the TLR4 ligand. Human (THP-1) (A) and chicken (HD-11) (B) macrophages stably transfected with the NF-κB luciferase reporter gene were coincubated with recombinant *C. jejuni* FlaC (100 ng), *Salmonella* FliC (50 ng), or *E. coli* LPS (25 ng) for 3 h, and NF-κB activation was measured in a SteadyGlo luciferase assay. Residual activation of the cells in all wells was determined by a luciferase measurement 19 h after the initial incubation. To analyze the reactivation potential, cells were preincubated with recombinant *C. jejuni* FlaC, *Salmonella* FliC, or *E. coli* LPS for 19 h and then coincubated with *E. coli* TLR4 ligand LPS (25 ng). The resulting reactivation potential of NF-κB was measured 3 h after the secondary coincubation step. As a control for TLR4-specific activity, control wells were preincubated with polymyxin B (10 µg/ml) 1 h before the initial activation, as indicated below the graph. For all measurements, relative luciferase activity is depicted as the percentage of maximal activation by LPS, which was defined as 100%. +, addition of substance on day 1; −, no addition of substance on day 1; *, addition of *E. coli* LPS on day 2. Significant *P* values are indicated by asterisks (Student’s *t* test, unpaired, two-sided), as follows: ***, *P* ≤ 0.001.

TLRs cooperate, synergize, and cross talk with each other and with signaling induced by other pattern recognition receptors, e.g., the cytoplasmic NOD-like receptor family, in the case of simultaneous or time-shifted stimulation (41, 42). For instance, it was demonstrated that TLR4 is downregulated by pretreating cells with ligands of other TLRs (43) and that TLR5 is upregulated by TLR1/2-mediated signaling (44). In addition, cross-tolerance to different TLR ligands, including TLR4, can be induced by prior TLR stimulation through a TLR downstream microRNA (miRNA)-mediated silencing mechanism of interleukin-1 receptor-associated kinase (IRAK) molecules (45–47). In order to investigate the influence of *C. jejuni* FlaC on other TLR-signaling pathways, we performed time-shifted coincubation experiments with different TLR ligands using stably transfected human THP-1 and chicken HD-11 cells (Fig. 6A and B). These assays involved a primary activation step and, on the second day, a secondary stimulation with *E. coli* ultrapure LPS (TLR4 ligand). As expected, THP-1 cells showed an enhanced NF-κB activation 3 h after primary coincubation with *E. coli* LPS and with *Salmonella* FliC (canonical TLR5 ligand) (Fig. 6A). Purified FlaC alone slightly (nonsignificantly) stimulated NF-κB in the initial activation experiment in both cell lines (Fig. 6A and B). In each case, the activation by all ligands was almost completely reversed 19 h after the initial coincubation, as expected (Fig. 6A and B). Only cells which we initially coincubated with LPS showed a slight residual NF-κB activation at the 19-h coincubation time point. Interestingly, preincubation for 19 h with FlaC (primary stimulus) was able to antagonize the activating effect of bacterial LPS (secondary stimulus) in these cells to an extent comparable with that of the canonical TLR ligands FliC and LPS, which were used as controls (Fig. 6A). Similar results were obtained using chicken HD-11 cells (Fig. 6B). In order to verify whether the inhibition of activation was not due to a direct competitive effect of FlaC for binding to TLR4, we also carried out coincubation assays with FlaC and LPS simultaneously. In this setting, FlaC only slightly inhibited LPS-mediated TLR activation (data not shown). LPS antagonist (polymyxin B) treatment of cells prevented primary LPS activation and the cross-tolerizing effect by FlaC (Fig. 6A).

### Live *C. jejuni* bacteria induce differential activation of cells in the presence or absence of FlaC.

**FIG 7:**
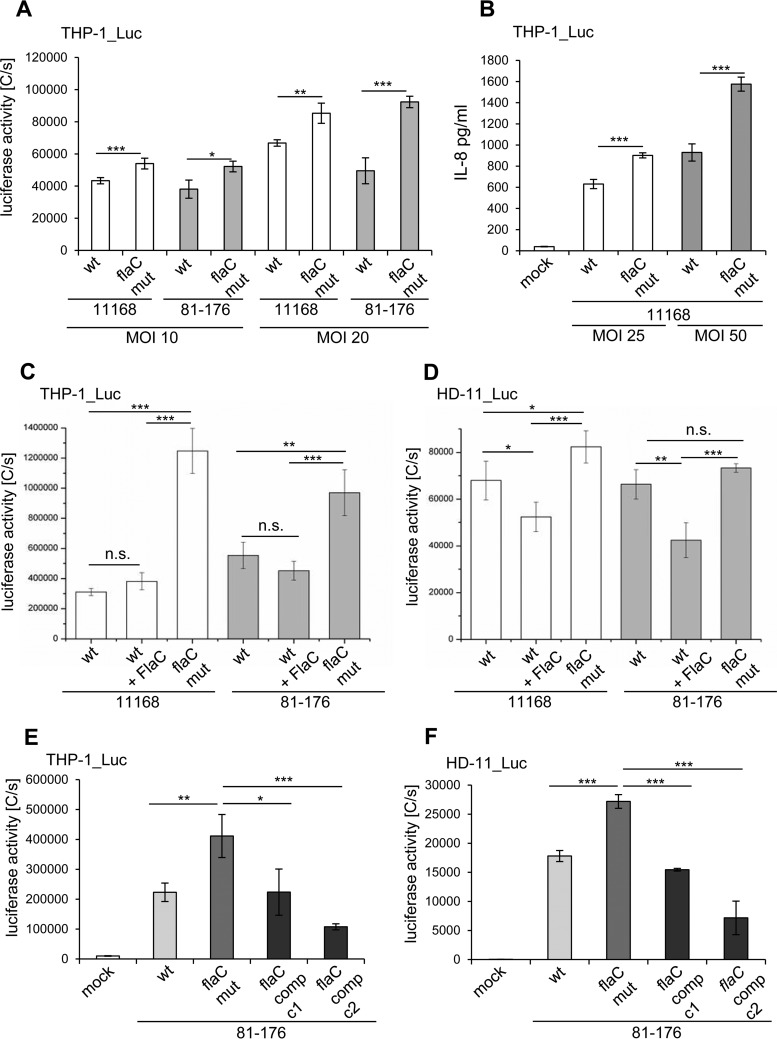
*C. jejuni flaC* mutants induce increased cell activation in human and chicken cells. (A and B) Comparison of cell activation of live *C. jejuni* with the respective *flaC* mutants of two strains, 11168 and 81-176. (A) Human THP-1 NF-κB luciferase reporter cells were coincubated with live *C. jejuni* bacteria of strain 11168 or 81-176 (wt) and corresponding isogenic *flaC* mutants (flaC mut) at different multiplicities of infection (MOI) for 3 h. NF-κB activation was determined using SteadyGlo luciferase substrate. Means and standard deviations from technical quadruplicates are shown as luciferase activity in counts per second (C/s). (B) IL-8 secretion induced by the live *C. jejuni* wild type and *flaC* mutants was measured by IL-8 ELISA. The statistical significance of differences was determined using Student’s *t* test (unpaired, one-sided). ***, *P* ≤ 0.001; **, 0.001 ≤ *P* ≤ 0.01; *, 0.01 ≤ *P* ≤ 0.05. (C and D) Human (THP-1) (C) and chicken (HD-11) (D) NF-κB luciferase reporter cell lines were coincubated with cleared lysate fractions of wild-type bacteria (wt) and corresponding *flaC* mutants (flaC mut) (100 ng of cleared lysate) of two different *C. jejuni* strains (11168, 81-176) for 3 h. For comparison, cells which were preincubated for 1 h with purified FlaC (100 ng) were also incubated with soluble fractions of wild-type lysates (100 ng) for 3 h. Luciferase activities were measured in a SteadyGlo luciferase assay. Mean values and standard deviations of triplicate measurements are depicted as luciferase activities (counts per second [C/s])*.* n.s., not significant. (E and F) Complementation of *flaC* restores the cell activation level by *C. jejuni*. (E) Human (THP-1) and (F) chicken (HD-11) macrophages stably transfected for the NF-κB luciferase reporter gene were coincubated for 3 h with soluble lysate fractions (100 ng) of the *C. jejuni* parental strain (wt), corresponding *flaC* mutant (flaC mut), and two *flaC* complementation clones of strain 81-176 (flaC comp, c1, and c2). NF-κB activation was measured using SteadyGlo substrate. Means and standard deviations from technical quadruplicates are shown as luciferase activity in counts per second [C/s]. Statistical significance was determined using Student’s *t* test (unpaired, two-sided). Significant *P* values are indicated by asterisks, as follows: *, 0.01 ≤ *P* ≤ 0.05; **, 0.001 ≤ *P* ≤ 0.01; ***, *P* ≤ 0.001.

As isolated FlaC can activate cells, the question of how this capacity and the presence of FlaC produced by live bacteria impact the complex interactions between bacteria and cells remained to be answered. To address this question, we coincubated live bacteria of two different *C. jejuni* parental strains, 11168 and 81-176, and their isogenic *flaC* mutants with THP-1 reporter cells at different multiplicities of infection (MOI). Cells were differentially activated in a dose-dependent manner (MOI dependent) by wild-type or *flaC* bacteria (Fig. 7A) of both strains. At early time points of coincubation relevant for primary innate immune responses (up to 4 h), *flaC* mutants induced significantly more NF-kB activation at all tested MOI than the wild type. The same effect was observed for the induction of IL-8 secretion by live *C. jejuni* bacteria in THP-1 cells (Fig. 7B).

In a different setting, we addressed the effect of FlaC more specifically by preincubating cells with highly purified FlaC before the addition of *C. jejuni* bacterial lysates. FlaC-supplemented wild-type lysates induced significantly less NF-kB activation even than the wild-type lysates alone, in particular in chicken HD-11 cells (Fig. 7D). In contrast, *flaC* mutant lysates of both *C. jejuni* strains displayed a significantly increased potency toward inducing NF-κB-dependent signaling in comparison to that of the wild-type lysates (Fig. 7C and D). Both cell lines were highly responsive to either TLR4 (Fig. 6A and B) or TLR2 (data not shown) ligands, suggesting that the activation by *C. jejuni* can be mediated by different bacterial agonists of Toll-like receptors, which may then be antagonized by FlaC. In comparison to the *flaC* mutant, a *flaC*-complemented strain expressing *flaC* under the control of its intrinsic promoter inserted into the *C. jejuni rdxA* gene locus (see Materials and Methods) was able again to reduce the response of THP-1 and HD-11 macrophage-like cells to *C. jejuni* whole bacterial lysates to the level induced by the parental strain or even below that level, an effect significantly different from that of the *flaC* mutant (Fig. 7E and F).

### *C. jejuni* FlaC acts on the chicken cecal immune response and alters microbiota composition in the chicken cecum.

**FIG 8:**
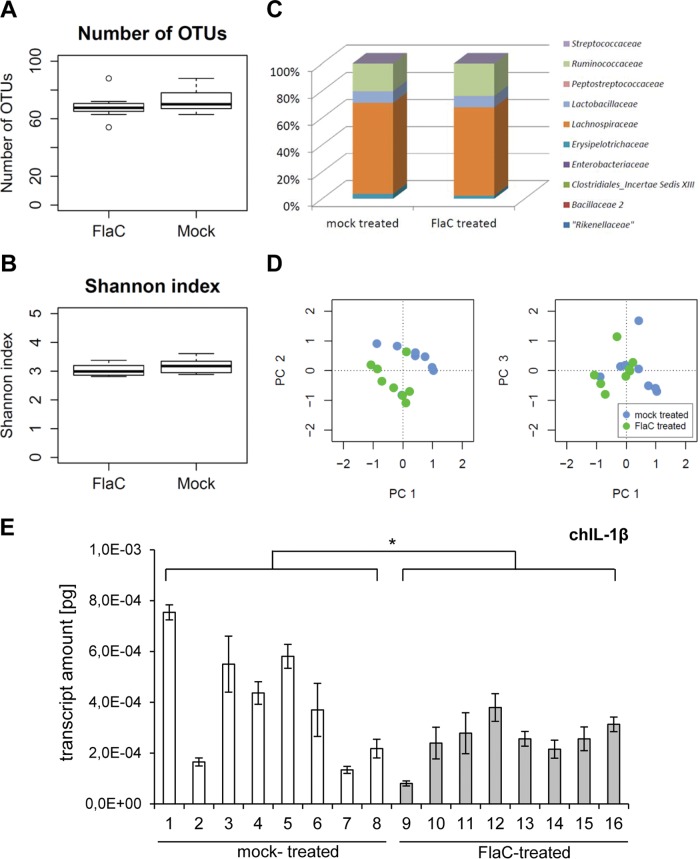
FlaC has a significant influence on the chicken cecal microbiota and cecal expression of chicken IL-1β. Microbiota analysis of chicken cecal tissue was performed using 16S rRNA gene amplicon sequencing. The microbiotas were compared between a chicken group (8 animals) of FlaC-treated animals and 8 animals that were mock (PBS) treated. Shown are comparative numbers of OTU (A), a comparison of the within-sample OTU diversity between the two groups (Shannon index) (B), and the comparative family assignments of OTU present in the cecal microbiotas of the two groups (C)**.** (D) A principal-coordinates analysis (PC; based on Bray-Curtis distances [see Text S1 in the supplemental material]) of the microbiota data indicated that the chickens in the FlaC-treated group (neon-green dots) have a significantly different microbiota composition than the chickens in the mock-treated group (light-blue dots). Depicted in the graphs are the distances between the microbiota data in the first three dimensions (axes PC 1 to PC 3) of the Bray-Curtis principal-coordinates analysis (distances between PC 1 and PC 2 in the left panel; distances between PC 1 and PC 3 in the right panel), of which the first axis (PC 1) explains 30.2% of the total variance in the data set, the second axis (PC 2) 26.4%, and the third axis (PC 3) 15.0%. For specific differences in the OTU assignments between the groups, see Table 2. (E) Results of a quantitative RT-PCR of chIL-1β mRNA in cecal tissue of mock- and FlaC-treated chickens. Transcript values were normalized against those of the chicken GAPDH transcript and are presented as absolute specific transcript amounts in picograms (2 µl of cDNA was used for each sample). Mean values and standard deviations from quadruplicate measurements are shown. The significance of difference (Student’s *t* test, unpaired, one-sided) in cytokine expression between both groups is indicated by an asterisk (*, *P* ≤ 0.05).

We designed an experiment to verify that purified FlaC is able to act in a potentially homeostatic manner on the microbiota in the ceca of live chicken. Two groups of 2-week-old SPF chickens that had been hatched and raised together were separated at the zero time point into two different cages. One FlaC-treated group received microgram amounts of highly purified FlaC (25 µg in cell culture-grade phosphate-buffered saline [PBS]) at three different time points (*t* = 0, *t* = 7 days, *t* = 10 days) by the intracloacal route to the colonic-cecal junction of the birds. The second group of birds was mock treated with sterile PBS at the same time points. At 14 days after the first treatment, the birds were necropsied and the cecal tissue was subjected to histopathological assessment, microbiota analysis, and real-time PCR analysis of chicken cytokine transcript levels in the ceca. Histopathology assessment showed comparable, low inflammatory scores from the cecal tissues of both groups (data not shown). Comparative microbiota analyses of the ceca between the two groups of birds revealed significant differences in the overall microbiota compositions (beta-diversity) (for the analysis of molecular variance [AMOVA] based on Bray-Curtis distances, *P* was 0.022; for the AMOVA based on Jaccard distances, *P* was 0.004) (Fig. 8D). The overall numbers of detected operative taxonomic units (OTU), their levels of overall diversity, and the abundances of different bacteria at the family level did not differ significantly between the microbiotas of the two groups (Fig. 8A to C), while single bacterial OTU differed significantly between both groups (Fig. 8 D; Table 2). Of the different chicken cytokines that we quantitated in real-time PCR (chIL-8, chK60, chK203, chIL-17a, chIL-1β), levels of chIL-1β were significantly different in the cecal tissue of both groups (significantly lower in the FlaC-treated group) (Fig. 8E).

10.1128/mSphere.00028-15.8 Text S1  Microbiota analysis of chicken cecum tissue. Download Text S1, PDF file, 0.02 MB. Copyright © 2015 Faber et al. 2016 Faber et al. This content is distributed under the terms of the Creative Commons Attribution 4.0 International license.

**TABLE 2:** Designation of group-specific OTU significantly different between microbiotas of FlaC-treated and mock-treated chickens

Specificity and OTU^a^	Class or order	Family	Genus (cluster)	Most closely related sequence (% nucleotide identity)
Mock-treated group				
OTU 63	*Erysipelotrichales*	*Erysipelotrichaceae*	*Erysipelatoclostridium*	*Clostridium innocuum* (93)
OTU 73	*Lactobacillales*	*Lactobacillaceae*	*Lactobacillus*	*Lactobacillus ingluviei* (98)
OTU 87	*Clostridiales*	*Clostridiaceae*	*Clostridium* (cluster IV)	*Clostridium* sp. strain YIT12069 (100)
OTU 134	*Clostridiales*	*Oscillospiraceae*	*Oscillibacter*	ND
FlaC-treated group				
OTU 50	*Erysipelotrichales*	*Erysipelotrichaceae*	*Clostridium* (cluster XVIII)	ND
OTU 52	*Clostridiales*	*Lachnospiraceae*	*Blautia*	*Blautia faecis* (*98*)
OTU 121	*Clostridiales*	*Lachnospiraceae*	*Blautia*	*Blautia hydrogenotrophica* (97)

a Designation of group-specific OTU at the genus or species level was verified by nucleotide BLAST search of 16S rRNA gene amplicon sequences using the NCBI nr database or SINA alignment with sequences listed in the Silva, Living Tree Project (LTP), or Ribosomal Database Project (RDP) database (http://www.arb-silva.de). Noted identity scores (percentages) reflect similarities of OTU-specific consensus nucleotide sequences with the identified closest bacterial relatives. ND, not determined due to low similarity (<90%) of database hits.

## DISCUSSION

*C. jejuni* and related bacteria have an unusual life style, since they are able to colonize many different host species (48–50) but thrive quite exclusively in one defined body niche, for example in the intestinal tracts of various host species. Therefore, it is to be expected that they (i) possess general features that allow them to modulate the immune responses of different host species and, at the same time, (ii) exhibit specific properties that facilitate the colonization of the intestinal habitat specifically. The innate immune response in the mature intestinal tract at the mucosal level is usually characterized by homeostatic downmodulation of TLR4- and TLR2-mediated responses (51–55) and an important continuous functional role of the innate flagellin receptor TLR5 (23). The intestine has several other unique characteristics important for pathogen colonization, e.g., a high density of resident microbiota of various species, a specifically primed immunological environment, and a specific availability of nutrients that is influenced by the microbiota (55). Little is known about how *C. jejuni* and closely related intestinal species interact with the innate immune systems of their hosts and with the major pattern recognition receptors (PRRs) of the TLR and NOD families. Glycans of the species appear to have modulatory effects (56), and it has been reported that the outer structural subunits of *C. jejuni* flagella, in particular the major flagellin FlaA, have a weak intrinsic ability to activate the innate immune system (15, 16). It has been hypothesized that the evasion of TLR5-mediated responses by the major *Campylobacter* flagellin FlaA, similar to that in the related species *Helicobacter pylori* (13, 14), may help the bacteria to achieve chronic infection in certain hosts by limiting the intestinal proinflammatory immune response. In contrast to primarily sterile body sites, each bacterial species resident in the intestine possesses a differential potential to elicit proinflammatory or homeostatic signaling (55, 57).

We have now identified a novel function of the *C. jejuni* flagellin-like secreted protein FlaC to modulate the host innate immune system. FlaC was previously described to be potentially involved in host-pathogen interactions (7), but its effects have not been known in much detail. FlaC was previously assigned to the group of potential *C. jejuni* virulence factors that are secreted with the help of the flagellar type III secretion system (5, 6). We were initially intrigued by the primary amino acid sequence of FlaC, which is very closely related to those of other flagellins but lacks most of the central D2 and D3 portions that are present in all known motility-associated flagellins of *Epsilonproteobacteria* (15, 58, 59). Although others had reported before that FlaC was not involved in the motility functions of *C. jejuni* flagella, we intensively tested the *flaC* mutants generated in two different *C. jejuni* strains for a loss of motility functions in tracking assays (not shown) and in motility plate assays. Corroborating previously published results (7), not the slightest reduction in the motility properties of *flaC* mutants could be identified, suggesting that FlaC is indeed not involved in motility functions but might serve another, yet-unknown function. With two different *C. jejuni* reference strains, we also experimentally confirmed previous results with an unrelated strain (7), showing that FlaC is predominantly a secreted protein. This further supported the hypothesis that FlaC does not act primarily in a bacterium-bound fashion and raised the question of which type of function FlaC could fulfill as a secreted protein in the natural habitat of *C. jejuni*.

*flaC* was found to be present in all tested strains of *C. jejuni*. After a search of genomic databases, *flaC* orthologues were clearly identified in a number of different *Campylobacter* species’ genomes, including *C. jejuni* and *C. coli*, in *W. succinogenes* (60), and in three enterohepatic, flagellated nonsheathed (61–63) *Helicobacter* species, *H. canadensis*, *H. pullorum*, and *H. winghamensis*. The *flaC*-positive species comprise the newly emerging intestinal human pathogens *Campylobacter concisus* (64), *H. canadensis*, *H. pullorum*, and *H. winghamensis* (65). Interestingly, *flaC* was present only in genomes of *Epsilonproteobacteria* species not possessing a flagellar sheath, which suggests that it has specifically evolved based on its potential to be secreted from the flagellar T3SS apparatus. Generating amino acid alignments of *C. jejuni* FlaC with a number of different flagellin protein sequences from various species, we sought to unravel sequence signatures in FlaC that might reveal something about its potential function.

Remarkably, FlaC amino acid sequences from different *Campylobacter* and *Helicobacter* species appeared to be chimeras between primary sequences of flagellins that had earlier been reported to activate the innate immune system via TLR5 (*Salmonella*, *Pseudomonas*) (9, 10) and sequences of other flagellins, mainly those of the *Epsilonproteobacteria*, that possess a low ability to elicit proinflammatory signaling via the TLR5 receptor (13–16). The conserved chimeric sequence nature of FlaCs was particularly obvious in the N-terminal flagellin D1 region (comprising amino acids 89 to 96) that has recently been assigned by structural analysis of a heterotetrameric complex between flagellin and the ectodomain of TLR5 to be directly involved in TLR5 binding as “primary binding interface B” (12). Galkin et al. had previously raised the hypothesis that the three-dimensional structure of the monomeric flagellins of nonstimulating bacterial species seemed to have evolved by changing its surface in a way that preserves multimeric flagellar assembly and motility yet prevents TLR5 activation (17). We obtained evidence under defined conditions *in vitro* that FlaC can bind to human TLR5. Structural studies of FlaC should clarify whether its fold and protein surface correspond to those of the activating or to the nonactivating flagellins and whether it is able at all to form multimers. Our bioinformatic analysis provided further evidence that, while the *flaC* gene is present and conserved over a wide range of intestinal species of *Epsilonproteobacteria*, it is not linked to a fixed gene neighborhood in the *flaC*-containing species. The function of the neighboring genes and their potential functional connection with FlaC are so far unknown.

In functional analyses, we sought to test the hypothesis that the FlaC flagellin might have evolved as an immunomodulatory protein. Since *C. jejuni* and other *Campylobacters* naturally colonize different hosts, we used cell lines of human and avian (chicken) origin for our analyses. FlaC activated the phosphorylation of MAP kinases p38 and ERK in human and chicken cell lines, which is a characteristic downstream signal of TLR5 activation (34, 38). Cytokine release in human cells coincubated with FlaC was almost negligible. Highly purified FlaC induced only minimal activation of NF-kB in both human and chicken cells but was able to activate MAPK-dependent cytokine transcript increases in human and chicken cells (both macrophage-like and epithelial cells). FlaC activation potential seemed to be partial or reduced in comparison to that of the canonical TLR5 agonist FliC. Chicken cells appeared to react more strongly to FlaC than human cells. One established property of TLR ligands is their proinflammatory property in acute infection or upon initial pathogen exposure (66, 67). FlaC appears to provide only a partial proinflammatory activation potential to cells, which is directed mainly toward MAP kinase activation and not toward NF-κB activation or cytokine release from cells. Thus, the FlaC signal might be primarily homeostatic and may lack certain features of other flagellins, which remain to be explored. This may be attributable to the chimeric flagellin sequence of FlaC or to other, yet-unknown features of FlaC. Since a high level of cellular cytokine release after TLR stimulation has been reported to be dependent on a combination of multiple signals (68, 69) provided by some flagellins, e.g., by the canonical TLR5 ligand *Salmonella* Typhimurium FliC, through the combined activation of TLR5 and the NLRC4 inflammasome (35) and possibly a third unknown signal (70, 71), one of the next approaches will be to clarify whether FlaC is capable of activating inflammasomes. Interestingly, it is just the region between amino acid residues 89 and 96 of flagellins, which was recently also shown to be also involved in flagellin adjuvanticity independently of TLR5 (71), that is highly conserved in different FlaCs and shared with immune-activating flagellins, such as FliC.

The secondary, mainly homeostatic, property of many TLR ligands (72), in addition to their proinflammatory characteristics, has been characterized as a primary TLR-mediated activation, followed by a quick downregulation of downstream signal transduction from several TLRs within the first 8 h after stimulation, which can be provided by microRNA-mediated feedback silencing mechanisms involving IRAK1 or IRAK4 (47, 73). Homeostatic signaling can be mediated for instance by TLR5-activating flagellins, such as *Salmonella* FliC (20). It has also been shown that prior TLR5 stimulation provides feedback inhibition against repeated TLR5 stimulation. Although not completely clarified, since only tumor necrosis factor alpha (TNF-α) production, and not the secretion of other cytokines, was tested as a readout in previous publications, TLR5 ligation, by upregulation of microRNAs, may also promote cross-tolerance development against the subsequent application of other TLR ligands (45, 46).

The property of cross-tolerization by FlaC may play a more important role in chronic colonization than acute infection and immune defense. This may be of particular relevance in the intestinal tract, where many other immune-activating or modulating stimuli produced by a wealth of commensals and resident microbiota are present (55, 57) and where also *Campylobacter* may provide other, proinflammatory signals to the host. Therefore, we tested the potential of purified *C. jejuni* FlaC to provide homeostatic signaling and cross-tolerance induction, which might facilitate *C. jejuni* persistence in the intestinal tracts of certain hosts *in vivo*. When FlaC was used as a first stimulus on human and chicken cells and the TLR4 ligand *E. coli* LPS was applied as a second stimulus, FlaC, similarly to FliC, prevented or downmodulated secondary stimulation. This downmodulation was time dependent and did not occur when FlaC and other non-TLR5 stimuli were applied at the same time, suggesting that FlaC indeed is able to provide a feedback mechanism against TLR stimulation. This finding suggests a mechanistic model by which FlaC might act predominantly as a homeostatic signal on TLR-positive cells and might thereby provide an advantage in long-term or persistent populations of the intestinal tract by *Campylobacter* and other gut-dwelling related species. This proposed mechanism was also supported by our assays in which cells were coincubated with live bacteria and lysates prepared from the *C. jejuni* wild type, *flaC* mutants, and a *flaC*-complemented strain. In these tests, *flaC* mutants led to a significantly increased stimulation of cells, in contrast to what occurred with the wild-type bacteria, or vice versa, the supplementation or complementation of FlaC decreased innate responses. Our data suggest that the activating potential of FlaC is higher toward chicken than toward human cells. It is not yet possible to clarify whether a host-specific difference regarding the downmodulation of proinflammatory signaling or feedback silencing mediated by *C. jejuni* FlaC exists.

*In vivo*, when we administered even very small amounts of ultrapure FlaC to the chicken intestine, this led to a significant modulation of the intestinal microbiota composition in the chicken cecum. This difference in composition, involving depletion or enrichment of various species of the *Lachnospiraceae* family and the genera *Clostridium*, *Lactobacillus*, *Oscillibacter*, and *Blautia*, was accompanied by a significant decrease in cytokine IL-1β expression in the ceca of the FlaC-treated birds in comparison to levels in the mock-treated group. The significant reduction in IL-1β expression in the chicken cecum by FlaC, which again suggests that FlaC promotes homeostatic or cross-tolerance effects in the intestinal environment, may be due either to a direct action of FlaC or to a combination of FlaC and the net outcome of an altered microbiota. The timing and causality of events and the host-specific action of FlaC, as well as *in vivo* infection experiments with *C. jejuni* (29), should be further addressed in chickens.

### Concluding remarks.

Interestingly, *C. jejuni* and other campylobacters cause chronic infection or colonization in avian, bovine, and porcine hosts; however, they only rarely become persistent in humans, where they lead to an acute, severe, and self-limiting infection. This study provided evidence that the *Campylobacter*-secreted flagellin FlaC, which is also present in other intestinal species, might be an important means of providing limited, and potentially homeostatic, immune activation in the intestinal tract. This activation may serve to modulate PRR-dependent responses that influence the interplay between the host innate immune system in the intestinal tract, the resident microbiota, and the sequence of events during acute or persistent *Campylobacter* infection. The question of whether FlaC plays a role in these two differential outcomes of host interaction by affecting TLR-dependent or other innate immune responses differently in different host species *in vivo* remains to be answered. In addition, both *C. jejuni* (74) and *S. enterica* (75) have recently been described to profit from a proinflammatory environment in the intestinal tract to successfully compete for nutrients with the resident microbiota, for instance by favoring the production and metabolic use of tetrathionate and other microbiota-derived metabolites. Since other microbe-associated molecular patterns of *C. jejuni* have evolved to be rather evasive toward innate immune recognition (15, 16, 76), the activation potential of FlaC, although apparently of a limited nature, may contribute to gaining a competitive edge in intestinal colonization and persistence by inducing both proinflammatory and homeostatic signals.

## MATERIALS AND METHODS

### Bacterial strains and culture conditions.

*Campylobacter jejuni* strains NCTC 11168 (ATCC 700819) and 81-176 (77) were used. *C. jejuni* cultures were grown at 37°C or 42°C under microaerobic conditions (10% CO_2_, 5% O_2_, 85% N_2_) in vented jars or under anaerobic conditions (10% CO_2_, 10% H_2_, 80% N_2_) in a Scholzen incubator on blood agar plates (blood agar base II; Oxoid, Wesel, Germany), supplemented with 10% defibrinated horse blood (Oxoid) and standard antibiotics (10 mg/liter vancomycin, 3.2 mg/liter polymyxin B, 5 mg/liter trimethoprim, 4 mg/liter amphotericin B), or in brain heart infusion broth (Oxoid) with the addition of 2.5 g/liter yeast extract (Merck, Darmstadt, Germany). The *C. jejuni* mutant strains were grown on blood agar plates with standard antibiotic supplement and 20 mg/liter chloramphenicol for selection. For coincubation assays with eukaryotic cells and for bacterial lysate preparations (ultrasonicated), bacteria were grown on plates for approximately 24 h before being harvested. *Escherichia coli* strains DH5α and MC1061 were used for cloning. For the overexpression of proteins, the *E. coli* strain BL21(DE3) was used. *E. coli* was cultured in Luria-Bertani (LB) broth (Difco LB agar; Lennox, BD Biosciences, Heidelberg, Germany) or on LB plates containing 1.5% Bacto agar. When appropriate, ampicillin (500 mg/liter) or kanamycin (20 mg/liter) was added to the medium.

### Cell types and culture conditions.

Different cell lines were used for cell coincubation and transfection assays. Human embryonic kidney 293 cells (HEK293T) were used for transfection and expression of recombinant proteins and cultured in Dulbecco’s minimal essential medium (MEM) (Biochrom, Berlin, Germany) supplemented with 10% (vol/vol) fetal bovine serum (FBS). The HD-11 chicken macrophage-like cell line and the human monocytic cell line THP-1 were maintained in Iscove’s basal medium (IBM) (Biochrom) supplemented with 10% (vol/vol) FBS. The Lovo human colon epithelial cell line (78) was cultured in Dulbecco’s MEM and Ham’s F-12 (1:1 mixture) (Biochrom) supplemented with 10% (vol/vol) FBS. HD-11, THP-1, and Lovo cell lines stably transfected with the firefly luciferase gene under the control of an NF-κB promoter were continuously cultured in the presence of puromycin (5 g/liter). All cell lines were routinely kept at 37°C in a 5% CO_2_ humidified atmosphere. Surface expression of TLR5 in THP-1 cells was verified by fluorescence-activated cell sorting (FACS) as previously published (79). Expression of *tlr5* mRNA in chicken HD-11 cells has been previously shown (80), and we have confirmed the presence of the *tlr5* transcript by semiquantitative reverse transcription-PCR (RT-PCR) in all cell lines used (not shown), except for Lovo cells. The functional activity of TLR5 was confirmed for all cell lines by ultrapure FliC stimulation and positive measurement of IL-8 secretion.

### Generation of *flaC* mutants by allelic exchange and complementation of *flaC*.

*flaC* mutants and *flaC* complementation strains in *C. jejuni* were generated by an allelic-exchange gene replacement strategy. Briefly, the *flaC* gene from strain 11168 was PCR amplified using primers CjflaC_F_BamHI and CjflaC_R_BamHI (Table 3) and cloned into pUC18. A unique internal BglII restriction site in the *flaC* gene was used to insert the chloramphenicol resistance cassette (CAT), derived from the plasmid pBHpC8 (81). The plasmid containing interrupted *flaC* was used for the natural transformation of two *C. jejuni* strains, 11168 and 81-176, and chloramphenicol-resistant clones were recovered after 3 days. The *flaC* mutant of the *C. jejuni* strain 81-176 was complemented by allelic gene replacement of *flaC* in the *rdxA* locus together with a gentamicin (Gm; aminoglycoside acetyltransferase, *aac*) resistance cassette from pUC1813*apra* (82). The *rdxA* gene from strain 81-176 was PCR amplified using primers CjrdxA_XbaI_F1 and CjrdxA_XbaI_R1 (Table 3) and cloned into pUC18 (Table 4). An internal BglII restriction site in the *rdxA* gene was used to insert *flaC* from strain 11168. This construct was amplified via inverse PCR using primers CjrdxA_SpeI_F3 and CjrdxA_ClaI_R3 (Table 3) and served for the insertion of the PCR-amplified Gm cassette (with primers Gm1_ClaI and Gm2_SpeI [Table 3]; template, the pUC1813*apra* plasmid) upstream of *flaC* using ClaI and SpeI restriction sites. The correct insertions after allelic exchange in CAT- or Gm-resistant clones recovered after natural transformation was confirmed by PCR using different primer combinations. The mutants and complementants showed no growth difference from the wild type in growth curve experiments (not shown). FlaC expression of the complementation strains was verified by Western blotting (not shown).

**TABLE 3:** Oligonucleotides used for gene amplification and cloning

Gene	Primers	Sequence (5′–3′)^a^	*T_m_* (°C)	Reference
*flaC* (Cj0720c)	CjflaC_F_BamHI	AAAGGATCCCAAAGTGGCTTAATGATGACG	55	This study
	CjflaC_R_BamHI	AAAGGATCCCGCTAGAGCTTGGACTTGAT	55	This study
	CjflaC_F3	AAAGGATCCATCTCTGATGCAACTATGATG	53	This study
	CjflaC_R2	AAACTCGAGTTGTAATAAATTAGCAATTTTGC	51	This study
*rdxA* (Cjj81176_1083)	CjrdxA_ClaI_R3	AAAATCGATGTTGATTGTAACATAGGGTTG	51	This study
	CjrdxA_SpeI_F3	AAAACTAGTCAAGTGCGAGTCATAATATC	51	This study
	CjrdxA_XbaI_F1	AAATCTAGAGTGATTTTGTCGTAGATGAAG	53	This study
	CjrdxA_XbaI_R1	AAATCTAGATATAAATTTCCAAGGTTCCA	51	This study
*SalTy* LT2 *fliC*	SalLT2FliCBamHI(F)	CGGGATCCATGGCACAAGTCATTAATAC	49	This study
	SalT2FliCEcoRI(R)	CGGAATTCCGCAGTAAAGAGAGGACG	51	This study
*chTLR5*	chTLR5_F1	AAAGGTACCGAGTCCGGATCCATGATGTTACAATCAA CGGCTAATAATTG	65	This study
	chTLR5_R1	AAAAGCGGCCGCCGTGTGAGACTGTCGCTATAGTTTG	69	This study
CAT cassette	pCAT1	AACAGCTATGACCATGATTAC	57	81
	pCAT2_BamHI	AGAGGATCCGATATCGCATGCCTGCAGAG	57	81
Gm cassette (*aac*)	Gm1_ClaI	AAAATCGATCGGGTGACTAACTAGGAGGAATAA	65	This study
	Gm2_SpeI	AGAACTAGTCCGTGTCATTATTCCCTCCAGGTA	67	This study

a Restriction sites in oligonucleotides are underlined. *T_m_*, melting temperature.

**TABLE 4:** Plasmids used in this study

Plasmid	Vector	Description	Resistance^a^	Reference or source
pEF6-hTLR5	pEF6-V5	Protein expression vector for human TLR5	Amp^r^	86
	pEF6-V5	Mammalian protein expression vector, originated from pEF6-TLR5-V5 by excision of the insert, EF-1α promoter, V5 tag	Amp^r^	13
pCJ801	pEF6-V5	Protein expression vector for chicken TLR5 (pEF6-chTLR5-V5)	Amp^r^	E. Faber and C. Josenhans, unpublished data
	pET28a	Protein expression vector, T7 *lac* promoter, 6×His tag, T7 tag	Km^r^	Novagen, Darmstadt, Germany
pCJ375	pET28a	*S. enterica* serovar Typhimurium LT2 *fliC* cloned into pET28a via BamHI and XhoI	Km^r^	S. K. Lee and C. Josenhans, unpublished data
pCJ1024	pET28a	*flaC* (*C. jejuni*, strain NCTC 11351) cloned into pET28a via BamHI and XhoI	Km^r^	This study
pCJ1025	pUC18	*flaC*::Cm (*C. jejuni* strain 11168)	Amp^r^ Cm^r^	This study
pCJ1405	pUC18	*rdxA::*Gm (*C. jejuni* strain 81-176) + *flaC* (*C. jejuni* strain 11168)	Amp^r^ Gm^r^	This study

a Amp^r^, ampicillin resistance; Km^r,^ kanamycin resistance; Cm^r^, chloramphenicol resistance; Gm^r^, gentamicin resistance.

### Plate motility assay.

The motility of *C. jejuni* wild-type strains and the corresponding *flaC* mutants and complemented strains was tested in a six-well plate format with semisolid medium, containing 2.8% (wt/vol) brucella broth (BD Biosciences), 0.3% (wt/vol) Bacto Agar (BD Biosciences), 5% (vol/vol) heat-inactivated horse serum (Gibco, Darmstadt, Germany), and standard antibiotics (see above). Bacteria were grown overnight on blood agar plates, adjusted to a final optical density at 600 nm (OD_600_) of 1.00, and inoculated with a pipette tip into the center of motility agar wells. The plates were then cultured for 2 days at 37°C under microaerobic or anaerobic conditions in a Scholzen incubator, and the swim diameter in the agar after the incubation was measured in comparison to a negative control (*C. jejuni flgR* mutant).

### DNA and protein methods.

DNA methods were performed according to standard protocols (83) using enzymes produced by New England Biolabs (NEB, Ipswich, NJ), Invitrogen (Carlsbad, California), or Roche (Basel, Switzerland). DNA from bacteria or tissue was prepared using the Qiagen Tissue Amp kit according to the manufacturers’ instructions, with slight modifications. PCRs were performed using *Taq* polymerase (Roche) or Phusion polymerase (NEB), if products with high amplification accuracy were required. Sanger sequencing technology was applied for sequencing of the cloned plasmids. Protein amounts were determined by bichinchoninic acid (BCA) protein assay (Thermo Scientific, Pierce, Rockford, IL), and protein analysis was performed by separation on denaturing 12% sodium dodecyl sulfate (SDS) polyacrylamide gels and Western immunoblot detection according to standard methods (84). Equal amounts of protein were loaded in each lane of the gels. Antibodies for labeling were used as indicated in the results. Visualization of immuno-reactive bands was obtained using the Super Signal West Pico chemiluminescent substrate (Pierce, Thermo Scientific, Bonn, Germany) and enhanced-chemiluminescence (ECL) hyperfilm (GE Healthcare, Piscataway, NJ). Analysis of phosphoproteins (P-p38, anti-P-p38 antibody, rabbit 92115; Cell Signalling Technology; used at a 1:1,000 dilution) of coincubated cells was performed on Western blots from cleared cell lysates prepared in radioimmunoprecipitation assay (RIPA) buffer (50 mM Tris-HCl, pH 7.5, 150 mM NaCl, 1% Triton X-100, 1 mM EDTA, 1 mM EGTA, protease inhibitor cocktail Complete [Roche], phosphatase inhibitor cocktail PhosStop [Roche]). Anti-p38 MAPK antibody (rabbit 92125; Cell Signalling Technology) was used on the same blots as a control for total p38. Quantification of chemiluminescent signals for the P-p38 blots was performed by densitometric measurements using the software ImageJ (http://rsb.info.nih.gov/ij/) and normalizing against signal intensities in each lane obtained using the p38 antibody. Additional normalization was performed against the corresponding loading controls visualized with the aid of anti-actin antibody (Chemicon MAB1501; mouse monoclonal antibody; used at a 1:2,000 dilution). In the case of sequential antibody application, membranes were stripped with Restore Western blot stripping buffer (Thermo Scientific, Pierce).

### Lysis and fractionation of *C. jejuni*.

*C. jejuni* lysates were generated by resuspending bacteria grown on blood agar plates in an appropriate volume of 0.9% NaCl and lysing by sonication (Branson Sonifier 450). To separate the soluble (cytoplasmic) and insoluble (membrane-associated) fractions of bacteria, *C. jejuni* lysates were centrifuged for 20 min at 9,000 × *g* and 4°C. The pellet, enriched for membrane-associated proteins, was resuspended in an appropriate volume of 0.9% NaCl; the supernatant represented the soluble bacterial fraction. For the preparation of secreted proteins, *C. jejuni* was grown in liquid culture to a defined OD_600_ of 0.4 to 0.6. Bacteria were harvested by centrifugation for 20 min at 9,000 × *g*. Subsequently, the supernatant, which contains secreted proteins, was filtered through a 0.22-µm-pore-size sterile filter (polyethersulfone [PES] membrane, Millipore Express; Millex GP, Schleicher & Schuell). Precipitation of secreted proteins was conducted with trichloroacetic acid (TCA) overnight at room temperature, followed by centrifugation for 15 min at 14,000 × *g* and 4°C and a final washing step with ice-cold 100% acetone. Secreted proteins were resuspended in modified SDS-PAGE loading buffer (20% [vol/vol] 5× SDS-PAGE loading buffer, 25% [vol/vol] Tris-HCl [1 M, pH = 8.0] in distilled water). For enriching surface-associated proteins, which contain, among others, flagellar proteins, *C. jejuni* was grown on blood agar plates for 2 to 3 days. Bacteria were resuspended in 0.9% NaCl, and proteins were sheared off the bacterial surface by repeatedly (up to 30 times) pushing the suspension through a 23-gauge needle. Sheared-off material was separated from bacterial cells by differential centrifugation steps: 20 min at 9,000 × *g* and ultracentrifugation for 1 h at 40,000 × *g* (Beckman Optima100 ultracentrifuge) and 4°C. Surface-associated proteins were resuspended in Tris buffer (100 mM Tris-HCl, pH 7.5). All bacterial fractions were analyzed on Western blots using anti-FlaC antiserum and respective fractionation controls (not shown).

### Recombinant expression and purification of *C. jejuni* FlaC and *S. enterica* serovar Typhimurium FliC.

For recombinant expression of *C. jejuni* protein FlaC in *E. coli*, the FlaC expression plasmid pCJ1024 (Table 4) was constructed, using primers CjFlaC_F3 and CjFlaC_R2 (Table 3), and transformed into *E. coli* BL21(DE3). Expression of FlaC was induced at early exponential phase (OD_600_ = 0.6) by adding 0.5 mM isopropyl-d-thiogalactopyranoside (IPTG). Bacteria were harvested after they reached the stationary phase (4 to 5 h), resuspended in appropriate volumes of lysis buffer (50 mM Tris-HCl, 150 mM NaCl, 5 mM MgCl_2_), and lysed by sonication. To separate the soluble from the insoluble fraction, the whole-cell lysate was centrifuged for 20 min at 9,000 × *g* and 4°C. The recombinantly expressed FlaC contained 6×His tags on its C and N termini and was purified from the soluble bacterial fraction by affinity chromatography using Ni^2+^-nitrilotriacetic acid (NTA)-Sepharose beads (Qiagen, Hilden, Germany) under denaturing conditions. The purification procedure comprised several wash steps with buffer B (8 M urea, 100 mM NaH_2_PO_4_, 100 mM Tris-HCl, pH 8.0) and buffer C (6 M urea, 100 mM NaH_2_PO_4_, 20 mM Tris-HCl, pH 6.3), with decreasing pH. Elution of the protein was achieved by using buffers with further decreased pH: buffer D (6 M urea, 100 mM NaH_2_PO_4_, 20 mM Tris-HCl, pH 5.8) and buffer E (6 M urea, 100 mM NaH_2_PO_4_, 20 mM Tris-HCl, pH 4.5). The single fractions were analyzed by SDS-PAGE with respect to their FlaC content; selected elution fractions were pooled and dialyzed against at least three changes of PBS using a dialysis chamber (Slide-A-Lyzer, molecular weight cutoff [MWCO], 3,500; Thermo Scientific). For coincubation experiments of eukaryotic cells with purified FlaC, an additional purification step by elution of the protein from an SDS gel in an Electro Eluter (model 422; Bio-Rad, Munich, Germany) was performed. Eluted protein was again dialyzed several times against cell culture-grade PBS, using pyrogen-free microconcentrator tubes (MWCO, 10,000; Amicon filter devices; Millipore) for centrifugation at 8,000 × *g* and 4°C. Expression and purification of *Salmonella* flagellin FliC was performed accordingly. The purity and amounts of ultrapure recombinant flagellins were finally checked on silver- or Coomassie blue-stained SDS gels (Fig. S7). *Limulus* assays (*Limulus* amebocyte lysis [LAL] chromogenic endpoint assay; Cambrex) of recombinant flagellins prepared by this method did not detect LPS above the detection limit of 1 endotoxin unit (EU) per µg of protein (13), and the preparations can thus be considered virtually free of contaminations by other TLR ligands. Before coincubation with cells, flagellar preparations were diluted in 1× PBS (cell culture grade) and monomerized by ultrasonication for 1 min. In cell activation experiments, the protein nature of the activating principle (purified FlaC) was again verified by heat-treating the protein preparations as a control, which led to a complete loss of activity (not shown).

10.1128/mSphere.00028-15.9 Figure S7  Determination of protein amounts and purity of highly purified flagellins. Recombinantly expressed *C. jejuni* FlaC and *S*. Typhimurium FliC were purified by affinity chromatography on Ni-NTA agarose beads and additionally eluted from SDS gels. To analyze purity and determine final protein amounts, SDS gels using different amounts of highly purified flagellins and defined amounts of bovine serum albumin (BSA; 0.5 µg, 1 µg, 2 µg from left to right) as a protein standard were performed. (Left) Lane 1, 1 µg of FlaC; lane 2, empty; lane 3, 2 µg of FlaC; (right) lane 1, 1 µg FliC; lane 2, 2 µg of FliC. The gels were stained with Coomassie blue for the detection of protein and comparative quantitation of amounts. Download Figure S7, PDF file, 0.1 MB. Copyright © 2015 Faber et al. 2016 Faber et al. This content is distributed under the terms of the Creative Commons Attribution 4.0 International license.

### Generation of antiserum against *C. jejuni* FlaC.

An antiserum raised against the recombinantly expressed and purified FlaC from *C. jejuni* was generated in rabbits by repeated intradermal injection in combination with Freund’s adjuvant (BioGenes, Berlin, Germany).

### Anti-His tag pulldown assay.

To investigate protein-protein interactions, pulldown analysis was performed. Two milligrams of purified recombinant 6×His-FlaC–6×His per sample was incubated for 30 min at 4°C with a 20-µl slurry of Cobalt (Co^2+^) beads (Talon beads; BD Biosciences) under continuous mixing in RIPA buffer (50 mM Tris-HCl, pH 7.5, 150 mM NaCl, 1% Triton X-100, 1 mM EDTA, 1 mM EGTA, protease inhibitor cocktail Complete [Roche], phosphatase inhibitors). An incubation step with cleared lysates (soluble fraction) of TLR5-V5 plasmid-transfected HEK293T cells (200 µg of lysate per sample) was performed, followed by coincubation for 1 h at 4°C under continuous mixing. After unbound protein was removed by stringent wash steps with imidazole-containing buffer (60 to 80 mM imidazole in PBS), the beads were resuspended in appropriate volumes of 2.5× SDS-PAGE loading buffer, and protein interactions were analyzed on Western blots. Precipitated TLR5 was detected using an antibody against the C-terminal V5 epitope tag (mouse monoclonal R960-25; Invitrogen Life Technologies).

### Cell transfection with plasmid DNA and protein expression analysis.

HEK293T cells, which were seeded in 24-well plates, were used for transfection experiments at 80% confluence. One hour before transfection, the medium was changed to 0.5 ml OptiMEM medium (Gibco) containing 5% fetal calf serum (FCS). The plasmid of interest (0.4 mg), which was prepared with an endo-free plasmid midi-kit (Qiagen, Hilden, Germany), was transfected using Lipofectamine 2000 (Invitrogen) according to the manufacturer’s instructions. To allow expression of transfected constructs, cells were further incubated for 24 h to 48 h before being harvested. Protein expression was then analyzed by Western immunoblotting.

### Coincubation of eukaryotic cells with proteins or live *C. jejuni* strains.

For coincubation experiments with live *C. jejuni* strains or purified proteins, cells were seeded into 96 (4 × 10^4^ cells in 100 µl per well)-, 24 (2 × 10^5^ cells in 1 ml per well)-, or 6 (2 × 10^6^ cells in 3 ml per well)-well plates 24 h prior to the infection. Media were replaced 30 min before the coincubations. For bacterial coincubations, cells were infected with freshly resuspended bacteria at an MOI of 10, 20, 25, or 50 and synchronized by centrifugation (300 × *g*, room temperature, 10 min). Equal bacterial amounts at the endpoint of cell coincubation with live *C. jejuni* bacteria were verified by Western immunoblotting using an anti-*C. jejuni* antiserum produced in rabbits.

In order to test the activation potential of protein preparations, cells were coincubated with different amounts of sonicated proteins (ultrapure flagellins) for 3 to 4 h, and a 96-well plate format was used for determining NF-κB activation of stable luciferase reporter cells. NF-κB-mediated luciferase activation was measured using SteadyGlow firefly luciferase substrate (Promega Inc., Madison, WI) in a Victor3 Wallac 1420 microtiter plate luminometer (GE Healthcare). The 24-well plate format was chosen for transfection experiments or for the further quantitative analysis of cytokine secretion (IL-8) or P-p38 analysis after coincubation with ultrapure proteins, live bacteria, or bacterial lysates. Equal MOIs were used to compare live bacteria between different strains; for cell coincubation with bacterial lysates, equal protein amounts of each lysate (BCA assay) were used for the different strains.

For assaying TLR4 specificity, polymyxin B (Sigma Aldrich) was added to the cells 1 h prior to starting the further incubation steps. For the 2-day experiments assessing tolerization against cell activation by *E. coli* LPS (TLR4 ligand ultrapure grade, from *E. coli* O111:B4; List Biological Laboratories Inc., Campbell, CA), THP-1 or HD-11 cells (2 × 10^4^ per well) were incubated in 96-well plates. One day after being seeded, the cells were activated by adding proteins or activation controls, respectively. Three hours and 19 h after the primary activation, NF-kB-dependent luciferase activity was determined using Promega SteadyGlo luciferase buffer in selected wells. At this time point, ultrapure LPS was added as a secondary stimulus in the remaining experiment wells and incubated for a further 3-h period, followed by the last luciferase measurement. For all experiments and conditions involving LPS, the ultrapure LPS preparation was used. IL-8 in supernatants of cell activation experiments was routinely determined using a commercially available ELISA (IL-8 OptEIA ELISA system; Becton, Dickinson, Inc., BD Biosciences), according to the manufacturer’s instructions. Coincubation experiments using a 6-well plate format were performed for RNA preparations and real-time PCR analysis.

### RNA preparation and real-time PCR.

For RNA preparation, cell pellets were resuspended in Qiagen RNeasy lysis buffer containing β-mercaptoethanol and transferred to lysing matrix D tubes containing ceramic beads (MPbio, Germany). The cells were lysed in a bead beater machine (FastPrep; Bio101 Thermo Scientific, Bonn, Germany) for 45 s. The RNA was further purified using the Qiagen RNeasy minikit according to the manufacturer’s instructions. Total RNA was quantitated in a NanoDrop 1000 device (Thermo Scientific, Bonn, Germany), and equal amounts of each sample were subjected to DNase I treatment (Roche). The residual content of DNA was determined by PCR using chicken glyceraldehyde-3-phosphate dehydrogenase (GAPDH) primers for HD-11 cells and human GAPDH primers for THP-1 and Lovo cells. If necessary, the residual DNA was removed by a second DNase I treatment and subsequent RNeasy column cleanup. cDNA was prepared from equivalent amounts (1 µg) of total RNA using Superscript III reverse transcriptase (Invitrogen) and oligo(dT) primers (Invitrogen) according to the manufacturer’s instructions.

Quantitative RT-PCR was performed using SYBR green mix (Qiagen) on equal amounts of cDNA (1 to 2 µl) and normalized to the results of a corresponding GAPDH or 16S rRNA gene control reaction mixture prepared from the same cDNAs (primers used for RT-PCR are listed in Table S2 in the supplemental material). Reactions were carried out in a Bio-Rad thermocycler (Bio-Rad C1000/CFX96 combined system [Bio-Rad, Hercules, CA]). Cycling conditions were as follows: denaturation for 10 min at 95°C and amplification for 40 cycles at 95°C for 15 to 45 s, at 59°C (or optimized according to the calculated annealing temperature of the primer pairs) for 15 s, and at 72°C for 30 s. For quantification of DNA amounts, a serial standard was prepared from a purified PCR product of the gene of interest. Each reaction was performed in triplicate.

10.1128/mSphere.00028-15.10 Table S2  Oligonucleotides used for RT-PCR. Download Table S2, PDF file, 0.03 MB. Copyright © 2015 Faber et al. 2016 Faber et al. This content is distributed under the terms of the Creative Commons Attribution 4.0 International license.

### Experimental administration of purified FlaC into SPF chicken and microbiota analyses.

SPF eggs from white SPF Leghorn layer chickens were purchased from Valo BioMedia GmbH (Bremen, Germany). After hatching, birds were kept jointly in metal cages without bedding and supplied with conventional, autoclaved feed and water *ad libitum*. Two weeks after hatching, the birds were separated into two groups consisting of 8 animals each and further kept in two separate closed cages in the same room. At this time point, one group of birds received 25 µg of purified recombinant FlaC in 200 µl sterile cell culture-grade PBS via intracloacal administration. The mock-treated group was given 200 µl of only sterile PBS by the same route. The application of purified FlaC (25 µg) or PBS (control group) via the same route was repeated after 7 and 10 days. Fourteen days after the initial administration, birds were sacrificed and their organs (liver, cecal tonsils, and right-hand cecum after gentle removal of contents) collected for CFU determination of culturable bacteria. The complete left-side ceca and three different gut sections of each bird were cleared of contents and fixed in 4% paraformaldehyde (PFA) for histopathological analysis. Two tissue samples of the tips of each right-hand cecum were sampled and stored in RNA-Later (Qiagen) at −20°C for further RNA preparation and at −18°C for DNA isolation and subsequent microbiota analyses, respectively. Animal experiments were conducted according to the German animal protection laws and approved by the federal authorities (LAVES, Lower Saxony, Germany) under the administrative identification number 33.14-42502-04-13/1282. Microbiota analysis by 16S rRNA gene amplicon sequencing from total chicken cecal tip tissue DNA was performed using PCR products of primer pairs covering the bacterial 16S rRNA gene variable regions V1 to V3 on a Roche 454 GS FLX+ pyrosequencing platform (85). Microbiota sequence reads were analyzed as previously described, with modifications (85; detailed in Text S1 in the supplemental material).

### Nucleotide sequence accession number.

The microbiota data (16S rRNA gene sequences) are available under accession number PRJEB11550 (European Nucleotide Archives ENA at EMBL-EBI).

## References

[1] Nachamkin I, Yang XH, Stern NJ 1993 Role of *Campylobacter jejuni* flagella as colonization factors for three-day-old chicks: analysis with flagellar mutants. Appl Environ Microbiol 59:1269–1273. 8517729 10.1128/aem.59.5.1269-1273.1993 PMC182076

[2] Hendrixson DR, DiRita VJ 2004 Identification of *Campylobacter jejuni* genes involved in commensal colonization of the chick gastrointestinal tract. Mol Microbiol 52:471–484. doi:10.1111/j.1365-2958.2004.03988.x. 15066034

[3] Wassenaar TM, van der Zeijst BAM, Ayling R, Newell DG 1993 Colonization of chicks by motility mutants of *Campylobacter jejuni* demonstrates the importance of flagellin A expression. J Gen Microbiol 139:1171–1175. doi:10.1099/00221287-139-6-1171. 8360610

[4] Guerry P, Alm RA, Power ME, Logan SM, Trust TJ 1991 Role of two flagellin genes in *Campylobacter* motility. J Bacteriol 173:4757–4764. 1856171 10.1128/jb.173.15.4757-4764.1991 PMC208154

[5] Poly F, Ewing C, Goon S, Hickey TE, Rockabrand D, Majam G, Lee L, Phan J, Savarino NJ, Guerry P 2007 Heterogeneity of a *Campylobacter jejuni* protein that is secreted through the flagellar filament. Infect Immun 75:3859–3867. doi:10.1128/IAI.00159-07. 17517862 PMC1951984

[6] Konkel ME, Klena JD, Rivera-Amill V, Monteville MR, Biswas D, Raphael B, Mickelson J 2004 Secretion of virulence proteins from *Campylobacter jejuni* is dependent on a functional flagellar export apparatus. J Bacteriol 186:3296–3303. doi:10.1128/JB.186.11.3296-3303.2004. 15150214 PMC415756

[7] Song YC, Jin S, Louie H, Ng D, Lau R, Zhang Y, Weerasekera R, Al Rashid S, Ward LA, Der SD, Chan VL 2004 FlaC, a protein of *Campylobacter jejuni* TGH9011 (ATCC43431) secreted through the flagellar apparatus, binds epithelial cells and influences cell invasion. Mol Microbiol 53:541–553. doi:10.1111/j.1365-2958.2004.04175.x. 15228533

[8] Goon S, Ewing CP, Lorenzo M, Pattarini D, Majam G, Guerry P 2006 A sigma28-regulated nonflagella gene contributes to virulence of *Campylobacter jejuni* 81-176. Infect Immun 74:769–772. doi:10.1128/IAI.74.1.769-772.2006. 16369037 PMC1346654

[9] Hayashi F, Smith KD, Ozinsky A, Hawn TR, Yi EC, Goodlett DR, Eng JK, Akira S, Underhill DM, Aderem A 2001 The innate immune response to bacterial flagellin is mediated by Toll-like receptor 5. Nature 410:1099–1103. doi:10.1038/35074106. 11323673

[10] Gewirtz AT, Navas TA, Lyons S, Godowski PJ, Madara JL 2001 Cutting edge: bacterial flagellin activates basolaterally expressed TLR5 to induce epithelial proinflammatory gene expression. J Immunol 167:1882–1885. doi:10.4049/jimmunol.167.4.1882. 11489966

[11] Smith KD, Andersen-Nissen E, Hayashi F, Strobe K, Bergman MA, Barrett SLR, Cookson BT, Aderem A 2003 Toll-like receptor 5 recognizes a conserved site on flagellin required for protofilament formation and bacterial motility. Nat Immunol 4:1247–1253. doi:10.1038/ni1011. 14625549

[12] Yoon S, Kurnasov O, Natarajan V, Hong M, Gudkov AV, Osterman AL, Wilson IA 2012 Structural basis of TLR5-flagellin recognition and signaling. Science 335:859–864. doi:10.1126/science.1215584. 22344444 PMC3406927

[13] Lee SK, Stack A, Katzowitsch E, Aizawa SI, Suerbaum S, Josenhans C 2003 *Helicobacter pylori* flagellins have very low intrinsic activity to stimulate human gastric epithelial cells via TLR5. Microbes Infect 5:1345–1356. doi:10.1016/j.micinf.2003.09.018. 14670447

[14] Gewirtz A, Yu Y, Gewirtz A, Yu Y, Krishna U, Israel D, Lyons S, Peek R Jr 2004 *Helicobacter pylori* flagellin evades toll-like receptor 5-mediated innate immunity. J Infect Dis 189:1914–1920. doi:10.1086/386289. 15122529

[15] Andersen-Nissen E, Smith KD, Strobe KL, Barrett SLR, Cookson BT, Logan SM, Aderem A 2005 Evasion of Toll-like receptor 5 by flagellated bacteria. Proc Natl Acad Sci U S A 102:9247–9252. doi:10.1073/pnas.0502040102. 15956202 PMC1166605

[16] Watson RO, Galán JE 2005 Signal transduction in *Campylobacter jejuni*-induced cytokine production. Cell Microbiol 7:655–665. doi:10.1111/j.1462-5822.2004.00498.x. 15839895

[17] Galkin VE, Yu X, Bielnicki J, Heuser J, Ewing CP, Guerry P, Egelman EH 2008 Divergence of quaternary structures among bacterial flagellar filaments. Science 320:382–385. doi:10.1126/science.1155307. 18420936

[18] Feuillet V, Medjane S, Mondor I, Demaria O, Pagni PP, Galan JE, Flavell RA, Alexopoulou L 2006 Involvement of Toll-like receptor 5 in the recognition of flagellated bacteria. Proc Natl Acad Sci U S A 103:12487–12492. doi:10.1073/pnas.0605200103. 16891416 PMC1567905

[19] Cullender T, Chassaing B, Janzon A, Kumar K, Muller C, Werner J, Angenent L, Bell M, Hay A, Peterson D, Walter J, Vijay-Kumar M, Gewirtz A, Ley R 2013 Innate and adaptive immunity interact to quench microbiome flagellar motility in the gut. Cell Host Microbe 14:571–581. doi:10.1016/j.chom.2013.10.009. 24237702 PMC3920589

[20] Vijay-Kumar M, Gewirtz AT 2009 Flagellin: key target of mucosal innate immunity. Mucosal Immunol 2:197–205. doi:10.1038/mi.2009.9. 19242410

[21] Vijay-Kumar M, Aitken JD, Carvalho FA, Cullender TC, Mwangi S, Srinivasan S, Sitaraman SV, Knight R, Ley RE, Gewirtz AT 2010 Metabolic syndrome and altered gut microbiota in mice lacking Toll-like receptor 5. Science 328:228–231. doi:10.1126/science.1179721. 20203013 PMC4714868

[22] Vijay-Kumar M, Aitken JD, Kumar A, Neish AS, Uematsu S, Akira S, Gewirtz AT 2008 Toll-like receptor 5-deficient mice have dysregulated intestinal gene expression and nonspecific resistance to *Salmonella*-induced typhoid-like disease. Infect Immun 76:1276–1281. doi:10.1128/IAI.01491-07. 18195036 PMC2258833

[23] Carvalho F, Koren O, Goodrich J, Johansson MV, Nalbantoglu I, Aitken J, Su Y, Chassaing B, Walters W, González A, Clemente J, Cullender T, Barnich N, Darfeuille-Michaud A, Vijay-Kumar M, Knight R, Ley R, Gewirtz A 2012 Transient inability to manage proteobacteria promotes chronic gut inflammation in TLR5-deficient mice. Cell Host Microbe 12:139–152. doi:10.1016/j.chom.2012.07.004. 22863420 PMC4310462

[24] Mellits KH, Mullen J, Wand M, Armbruster G, Patel A, Connerton PL, Skelly M, Connerton IF 2002 Activation of the transcription factor NF-kappaB by *Campylobacter jejuni*. Microbiology 148:2753–2763. doi:10.1099/00221287-148-9-2753. 12213922

[25] Jones MA, Totemeyer S, Maskell DJ, Bryant CE, Barrow PA 2003 Induction of proinflammatory responses in the human monocytic cell line THP-1 by *Campylobacter jejuni*. Infect Immun 71:2626–2633. doi:10.1128/IAI.71.5.2626-2633.2003. 12704137 PMC153272

[26] Smith CK, Kaiser P, Rothwell L, Humphrey T, Barrow PA, Jones MA 2005 *Campylobacter jejuni*-induced cytokine responses in avian cells. Infect Immun 73:2094–2100. doi:10.1128/IAI.73.4.2094-2100.2005. 15784550 PMC1087459

[27] Borrmann E, Berndt A, Hänel I, Köhler H 2007 *Campylobacter*-induced interleukin-8 responses in human intestinal epithelial cells and primary intestinal chick cells. Vet Microbiol 124:115–124. doi:10.1016/j.vetmic.2007.04.041. 17540517

[28] Larson CL, Shah DH, Dhillon AS, Call DR, Ahn S, Haldorson GJ, Davitt C, Konkel ME 2008 *Campylobacter jejuni* invade chicken LMH cells inefficiently and stimulate differential expression of the chicken CXCLi1 and CXCLi2 cytokines. Microbiology 154:3835–3847. doi:10.1099/mic.0.2008/021279-0. 19047751

[29] De Zoete MR, Keestra AM, Roszczenko P, van Putten JPM 2010 Activation of human and chicken Toll-like receptors by *Campylobacter* spp. Infect Immun 78:1229–1238. doi:10.1128/IAI.00897-09. 20038539 PMC2825908

[30] Andersen-Nissen E, Smith KD, Bonneau R, Strong RK, Aderem A 2007 A conserved surface on Toll-like receptor 5 recognizes bacterial flagellin. J Exp Med 204:393–403. doi:10.1084/jem.20061400. 17283206 PMC2118731

[31] Keestra AM, de Zoete MR, van Aubel RAMH, van Putten JPM 2008 Functional characterization of chicken TLR5 reveals species-specific recognition of flagellin. Mol Immunol 45:1298–1307. doi:10.1016/j.molimm.2007.09.013. 17964652

[32] Keestra AM, van Putten JPM 2008 Unique properties of the chicken TLR4/MD-2 complex: selective lipopolysaccharide activation of the MyD88-dependent pathway. J Immunol 181:4354–4362. doi:10.4049/jimmunol.181.6.4354. 18768894

[33] De Zoete MR, Keestra AM, Wagenaar JA, van Putten JPM 2010 Reconstitution of a functional Toll-like receptor 5 binding site in *Campylobacter jejuni* flagellin. J Biol Chem 285:12149–12158. doi:10.1074/jbc.M109.070227. 20164175 PMC2852954

[34] Yu Y, Zeng H, Lyons S, Carlson A, Merlin D, Neish AS, Gewirtz AT 2003 TLR5-mediated activation of p38 MAPK regulates epithelial IL-8 expression via a post-transcriptional mechanism. Am J Physiol Gastrointest Liver Physiol 285:G282–G290. doi:10.1152/ajpgi.00503.2002. 12702497

[35] Miao EA, Andersen-Nissen E, Warren SE, Aderem A 2007 TLR5 and Ipaf: dual sensors of bacterial flagellin in the innate immune system. Semin Immunopathol 29:275–288. doi:10.1007/s00281-007-0078-z. 17690885

[36] Smedberg JR, Westcott MM, Ahmed M, Lyles DS 2014 Signaling pathways in murine dendritic cells that regulate the response to vesicular stomatitis virus vectors that express flagellin. J Virol 88:777–785. doi:10.1128/JVI.02898-13. 24198430 PMC3911629

[37] von Moltke J, Ayres JS, Kofoed EM, Chavarría-Smith J, Vance RE 2013 Recognition of bacteria by inflammasomes. Annu Rev Immunol 31:73–106. doi:10.1146/annurev-immunol-032712-095944. 23215645

[38] Zhang Z, Reenstra W, Weiner DJ, Louboutin J, Wilson JM 2007 The p38 mitogen-activated protein kinase signaling pathway is coupled to Toll-like receptor 5 to mediate gene regulation in response to *Pseudomonas aeruginosa* infection in human airway epithelial cells. Infect Immun 75:5985–5992. doi:10.1128/IAI.00678-07. 17908812 PMC2168327

[39] Staeheli P, Puehler F, Schneider K, Göbel TW, Kaspers B 2001 Cytokines of birds: conserved functions—a largely different look. J Interferon Cytokine Res 21:993–1010. doi:10.1089/107999001317205123. 11798457

[40] Macindoe G, Mavridis L, Venkatraman V, Devignes M, Ritchie DW 2010 HexServer: an FFT-based protein docking server powered by graphics processors. Nucleic Acids Res 38:W445–W449. doi:10.1093/nar/gkq311. 20444869 PMC2896144

[41] Begue B, Dumant C, Bambou JC, Beaulieu JF, Chamaillard M, Hugot JP, Goulet O, Schmitz J, Philpott DJ, Cerf-Bensussan N, Ruemmele FM 2006 Microbial induction of CARD15 expression in intestinal epithelial cells via Toll-like receptor 5 triggers an antibacterial response loop. J Cell Physiol 209:241–252. doi:10.1002/jcp.20739. 16897777

[42] Tukhvatulin AI, Gitlin II, Shcheblyakov DV, Artemicheva NM, Burdelya LG, Shmarov MM, Naroditsky BS, Gudkov AV, Gintsburg AL, Logunov DY 2013 Combined stimulation of Toll-like receptor 5 and NOD1 strongly potentiates activity of NF-kappaB, resulting in enhanced innate immune reactions and resistance to *Salmonella enterica* serovar Typhimurium infection. Infect Immun 81:3855–3864. doi:10.1128/IAI.00525-13. 23897616 PMC3811746

[43] Palazzo M, Gariboldi S, Zanobbio L, Dusio GF, Selleri S, Bedoni M, Balsari A, Rumio C 2008 Cross-talk among Toll-like receptors and their ligands. Int Immunol 20:709–718. doi:10.1093/intimm/dxn027. 18397908

[44] Koller B, Kappler M, Latzin P, Gaggar A, Schreiner M, Takyar S, Kormann M, Kabesch M, Roos D, Griese M, Hartl D 2008 TLR expression on neutrophils at the pulmonary site of infection: TLR1/TLR2-mediated up-regulation of TLR5 expression in cystic fibrosis lung disease. J Immunol 181:2753–2763. doi:10.4049/jimmunol.181.4.2753. 18684966

[45] Mizel SB, Snipes JA 2002 Gram-negative flagellin-induced self-tolerance is associated with a block in interleukin-1 receptor-associated kinase release from Toll-like receptor 5. J Biol Chem 277:22414–22420. doi:10.1074/jbc.M201762200. 11953430

[46] De Vos AF, Pater JM, van den Pangaart PS, de Kruif MD, van Veer C, van der Poll T 2009 *In vivo* lipopolysaccharide exposure of human blood leukocytes induces cross-tolerance to multiple TLR ligands. J Immunol 183:533–542. doi:10.4049/jimmunol.0802189. 19542464

[47] Nahid MA, Yao B, Dominguez-Gutierrez PR, Kesavalu L, Satoh M, Chan EKL 2013 Regulation of TLR2-mediated tolerance and cross-tolerance through IRAK4 modulation by miR-132 and miR-212. J Immunol 190:1250–1263. doi:10.4049/jimmunol.1103060. 23264652 PMC3552145

[48] Gripp E, Hlahla D, Didelot X, Kops F, Maurischat S, Tedin K, Alter T, Ellerbroek L, Schreiber K, Schomburg D, Janssen T, Bartholomäus P, Hofreuter D, Woltemate S, Uhr M, Brenneke B, Grüning P, Gerlach G, Wieler L, Suerbaum S, Josenhans C 2011 Closely related *Campylobacter jejuni* strains from different sources reveal a generalist rather than a specialist lifestyle. BMC Genomics 12:584. doi:10.1186/1471-2164-12-584. 22122991 PMC3283744

[49] Horrocks SM, Anderson RC, Nisbet DJ, Ricke SC 2009 Incidence and ecology of *Campylobacter jejuni* and *coli* in animals. Anaerobe 15:18–25. doi:10.1016/j.anaerobe.2008.09.001. 18849005

[50] Young TA, Davis LM, DiRita VJ 2007 *Campylobacter jejuni*: molecular biology and pathogenesis. Nat Rev Microbiol 5:665–679. doi:10.1016/j.jmb.2007.02.077. 17703225

[51] Cario E, Podolsky DK 2000 Differential alteration in intestinal epithelial cell expression of Toll-like receptor 3 (TLR3) and TLR4 in inflammatory bowel disease. Infect Immun 68:7010–7017. doi:10.1128/IAI.68.12.7010-7017.2000. 11083826 PMC97811

[52] Fukata M, Abreu MT 2009 Pathogen recognition receptors, cancer and inflammation in the gut. Curr Opin Pharmacol 9:680–687. doi:10.1016/j.coph.2009.09.006. 19828376 PMC2826797

[53] Furrie E, Macfarlane S, Thomson G, Macfarlane GT 2005 Toll-like receptors-2, -3 and -4 expression patterns on human colon and their regulation by mucosal-associated bacteria. Immunology 115:565–574. doi:10.1111/j.1365-2567.2005.02200.x. 16011525 PMC1782176

[54] Vamadevan AS, Fukata M, Arnold ET, Thomas LS, Hsu D, Abreu MT 2010 Regulation of Toll-like receptor 4-associated MD-2 in intestinal epithelial cells: a comprehensive analysis. Innate Immun 16:93–103. doi:10.1177/1753425909339231. 19710105 PMC2846239

[55] Carvalho FA, Aitken JD, Vijay-Kumar M, Gewirtz AT 2012 Toll-like receptor-gut microbiota interactions: perturb at your own risk! Annu Rev Physiol 74:177–198. doi:10.1146/annurev-physiol-020911-153330. 22035346

[56] Day CJ, Semchenko EA, Korolik V 2012 Glycoconjugates play a key role in *Campylobacter jejuni* infection: interactions between host and pathogen. Front Cell Infect Microbiol 2:9. doi:10.3389/fcimb.2012.00009. 22919601 PMC3417407

[57] Brown EM, Sadarangani M, Finlay BB 2013 The role of the immune system in governing host-microbe interactions in the intestine. Nat Immunol 14:660–667. doi:10.1038/ni.2611. 23778793

[58] Beatson SA, Minamino T, Pallen MJ 2006 Variation in bacterial flagellins: from sequence to structure. Trends Microbiol 14:151–155. doi:10.1016/j.tim.2006.02.008. 16540320

[59] Yonekura K, Maki-Yonekura S, Namba K 2003 Complete atomic model of the bacterial flagellar filament by electron cryomicroscopy. Nature 424:643–650. doi:10.1038/nature01830. 12904785

[60] Baar C, Eppinger M, Raddatz G, Simon J, Lanz C, Klimmek O, Nandakumar R, Gross R, Rosinus A, Keller H, Jagtap P, Linke B, Meyer F, Lederer H, Schuster SC 2003 Complete genome sequence and analysis of *Wolinella succinogenes*. Proc Natl Acad Sci U S A 100:11690–11695. doi:10.1073/pnas.1932838100. 14500908 PMC208819

[61] Melito PL, Munro C, Chipman PR, Woodward DL, Booth TF, Rodgers FG 2001 *Helicobacter winghamensis* sp. nov., a novel *Helicobacter* sp. isolated from patients with gastroenteritis. J Clin Microbiol 39:2412–2417. doi:10.1128/JCM.39.7.2412-2417.2001. 11427547 PMC88163

[62] Whary MT, Fox JG 2004 Natural and experimental *Helicobacter* infections. Comp Med 54:128–158. 15134359

[63] Solnick JV, Schauer DB 2001 Emergence of diverse *Helicobacter* species in the pathogenesis of gastric and enterohepatic diseases. Clin Microbiol Rev 14:59–97. doi:10.1128/CMR.14.1.59-97.2001. 11148003 PMC88962

[64] Kaakoush NO, Mitchell HM 2012 *Campylobacter concisus*—a new player in intestinal disease. Front Cell Infect Microbiol 2:4. doi:10.3389/fcimb.2012.00004. 22919596 PMC3417403

[65] Hansen R, Thomson JM, Fox JG, El-Omar EM, Hold GL 2011 Could *Helicobacter* organisms cause inflammatory bowel disease? FEMS Immunol Med Microbiol 61:1–14. doi:10.1111/j.1574-695X.2010.00744.x. 20955468

[66] Takeda K, Akira S 2005 Toll-like receptors in innate immunity. Int Immunol 17:1–14. doi:10.1093/intimm/dxh186. 15585605

[67] Sasai M, Yamamoto M 2013 Pathogen recognition receptors: ligands and signaling pathways by Toll-like receptors. Int Rev Immunol 32:116–133. doi:10.3109/08830185.2013.774391. 23570313

[68] Franchi L, Park JH, Shaw MH, Marina-Garcia N, Chen G, Kim YG, Nunez G 2008 Intracellular NOD-like receptors in innate immunity, infection and disease. Cell Microbiol 10:1–8. 17944960 10.1111/j.1462-5822.2007.01059.x

[69] Sanders CJ, Franchi L, Yarovinsky F, Uematsu S, Akira S, Núñez G, Gewirtz AT 2009 Induction of adaptive immunity by flagellin does not require robust activation of innate immunity. Eur J Immunol 39:359–371. doi:10.1002/eji.200838804. 19152336 PMC2734905

[70] Lopez-Yglesias AH, Zhao X, Quarles EK, Lai MA, Vandenbos T, Strong RK, Smith KD 2014 Flagellin induces antibody responses through a TLR5- and inflammasome-independent pathway. J Immunol 192:1587–1596. doi:10.4049/jimmunol.1301893. 24442437 PMC3925749

[71] Zhang L, Pan Z, Kang X, Yang Y, Kang H, Zhang N, Rosati JM, Jiao X 2015 Amino acids 89–96 of Salmonella typhimurium flagellin represent the major domain responsible for TLR5-independent adjuvanticity in the humoral immune response. Cell Mol Immunol 12:625–632. doi:10.1038/cmi.2014.76. 25195514 PMC4579647

[72] Rakoff-Nahoum S, Paglino J, Eslami-Varzaneh F, Edberg S, Medzhitov R 2004 Recognition of commensal microflora by Toll-like receptors is required for intestinal homeostasis. Cell 118:229–241. doi:10.1016/j.cell.2004.07.002. 15260992

[73] O’Neill LA, Sheedy FJ, McCoy CE 2011 MicroRNAs: the fine-tuners of Toll-like receptor signalling. Nat Rev Immunol 11:163–175. doi:10.1038/nri2957. 21331081

[74] Liu Y, Denkmann K, Kosciow K, Dahl C, Kelly DJ 2013 Tetrathionate stimulated growth of *Campylobacter jejuni* identifies a new type of bi-functional tetrathionate reductase (TsdA) that is widely distributed in bacteria. Mol Microbiol 88:173–188. doi:10.1111/mmi.12176. 23421726

[75] Winter SE, Thiennimitr P, Winter MG, Butler BP, Huseby DL, Crawford RW, Russell JM, Bevins CL, Adams LG, Tsolis RM, Roth JR, Bäumler AJ 2010 Gut inflammation provides a respiratory electron acceptor for *Salmonella*. Nature 467:426–429. doi:10.1038/nature09415. 20864996 PMC2946174

[76] van Mourik A, Steeghs L, van Laar J, Meiring HD, Hamstra HJ, van Putten JPM, Wosten MMSM 2010 Altered linkage of hydroxyacyl chains in lipid A of *Campylobacter jejuni* reduces TLR4 activation and antimicrobial resistance. J Biol Chem 285:15828–15836. doi:10.1074/jbc.M110.102061. 20351099 PMC2871450

[77] Korlath JA, Osterholm MT, Judy LA, Forfang JC, Robinson RA 1985 A point-source outbreak of campylobacteriosis associated with consumption of raw milk. J Infect Dis 152:592–596. doi:10.1093/infdis/152.3.592. 4031557

[78] Drewinko B, Romsdahl MM, Yang LY, Ahearn MJ, Trujillo JM 1976 Establishment of a human carcinoembryonic antigen-producing colon adenocarcinoma cell line. Cancer Res 36:467–475. 1260746

[79] Cho H, Choi E, Lee S, Kim K, Park S, Lee CK, Lee S 2011 All-trans retinoic acid induces TLR-5 expression and cell differentiation and promotes flagellin-mediated cell functions in human THP-1 cells. Immunol Lett 136:97–107. doi:10.1016/j.imlet.2011.01.001. 21237205

[80] Iqbal M, Philbin VJ, Smith AL 2005 Expression patterns of chicken Toll-like receptor mRNA in tissues, immune cell subsets and cell lines. Vet Immunol Immunopathol 104:117–127. doi:10.1016/j.vetimm.2004.11.003. 15661337

[81] Sterzenbach T, Bartonickova L, Behrens W, Brenneke B, Schulze J, Kops F, Chin EY, Katzowitsch E, Schauer DB, Fox JG, Suerbaum S, Josenhans C 2008 Role of the *Helicobacter hepaticus* flagellar sigma factor FliA in gene regulation and murine colonization. J Bacteriol 190:6398–6408. doi:10.1128/JB.00626-08. 18689480 PMC2566010

[82] Bury-Mone S, Skouloubris S, Dauga C, Thiberge J, Dailidiene D, Berg DE, Labigne A, de Reuse H 2003 Presence of active aliphatic amidases in *Helicobacter* species able to colonize the stomach. Infect Immun 71:5613–5622. doi:10.1128/IAI.71.10.5613-5622.2003. 14500481 PMC201111

[83] Sambrook J, Russell DG 2004 Molecular cloning: a laboratory manual. Cold Spring Harbor Laboratory Press, Cold Spring Harbor, NY.

[84] Towbin H, Staehelin T, Gordon J 1979 Electrophoretic transfer of proteins from polyacrylamide gels to nitrocellulose sheets: procedure and some applications. Proc Natl Acad Sci U S A 76:4350–4354. doi:10.1073/pnas.76.9.435083. 388439 PMC411572

[85] Yang I, Eibach D, Kops F, Brenneke B, Woltemate S, Schulze J, Bleich A, Gruber AD, Muthupalani S, Fox JG, Josenhans C, Suerbaum S 2013 Intestinal microbiota composition of interleukin-10 deficient C57BL/6J mice and susceptibility to *Helicobacter hepaticus*-induced colitis. PLoS One 8:e70783. doi:10.1371/journal.pone.0070783. 23951007 PMC3739778

[86] Smith MF Jr, Mitchell A, Li G, Ding S, Fitzmaurice AM, Ryan K, Crowe S, Goldberg JB 2003 Toll-like receptor (TLR) 2 and TLR5, but not TLR4, are required for *Helicobacter pylori*-induced NF-kappaB activation and chemokine expression by epithelial cells. J Biol Chem 278:32552–32560. doi:10.1074/jbc.M305536200. 12807870

