# Supplementary figures and images for: Novel Immunomodulatory Flagellin-Like Protein FlaC in Campylobacter jejuni and Other Campylobacterales
Source: mSphere. 2015 Dec 2;1(1):e00028-15. doi: 10.1128/mSphere.00028-15 (PMC4863622; doi:10.1128/mSphere.00028-15)

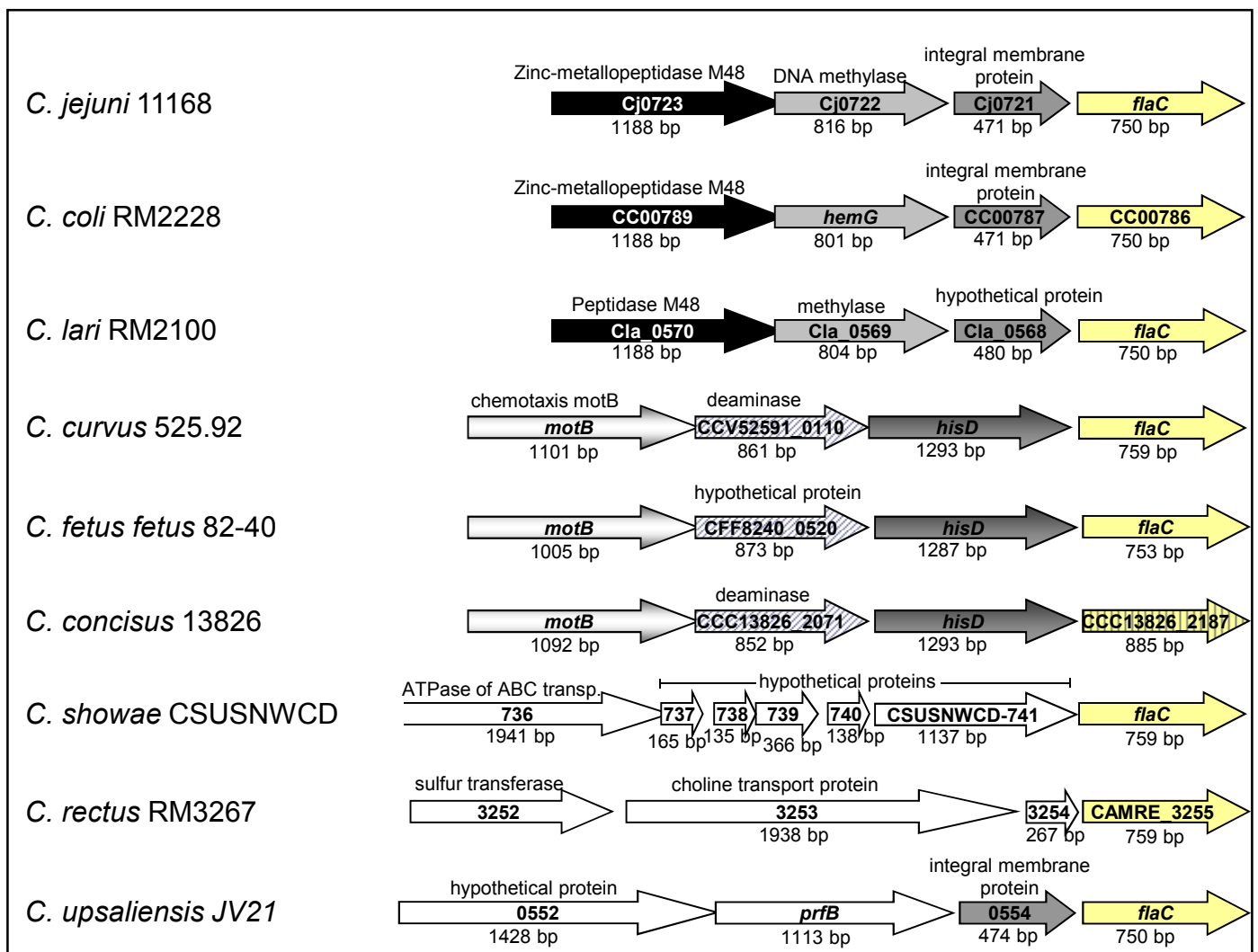

Fig. S1

Supplement: Figure S1 [file sph001160032sf4.pdf]

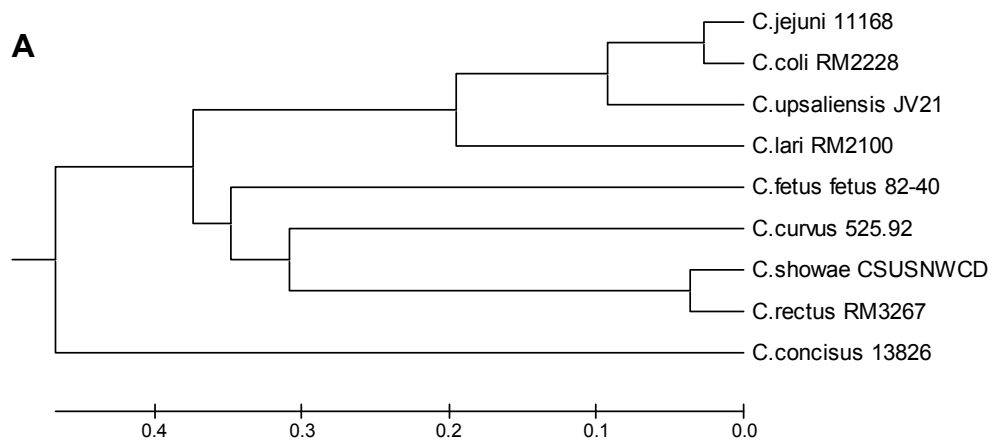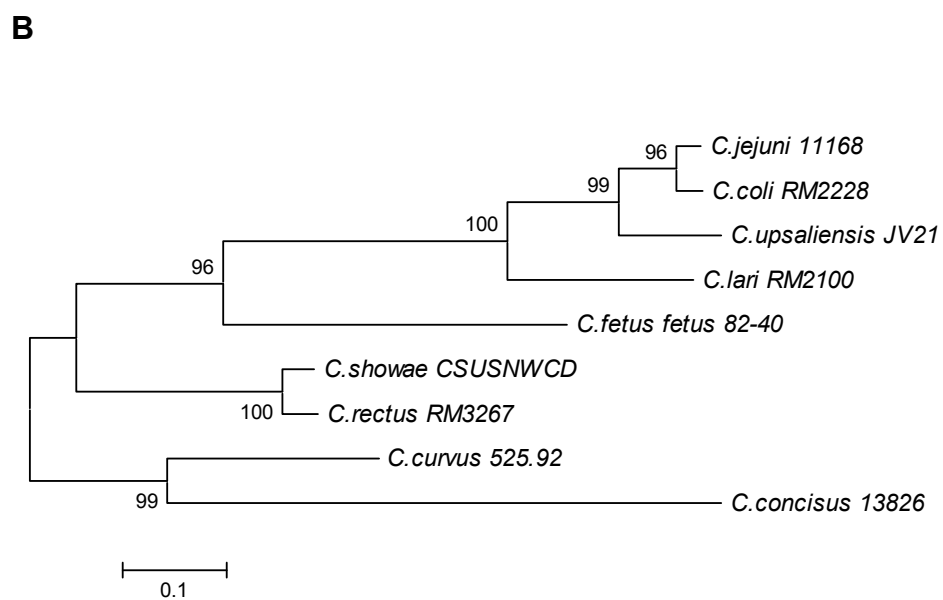

**Fig. S2**

Supplement: Figure S2 [file sph001160032sf5.pdf]

# *flaC* operon

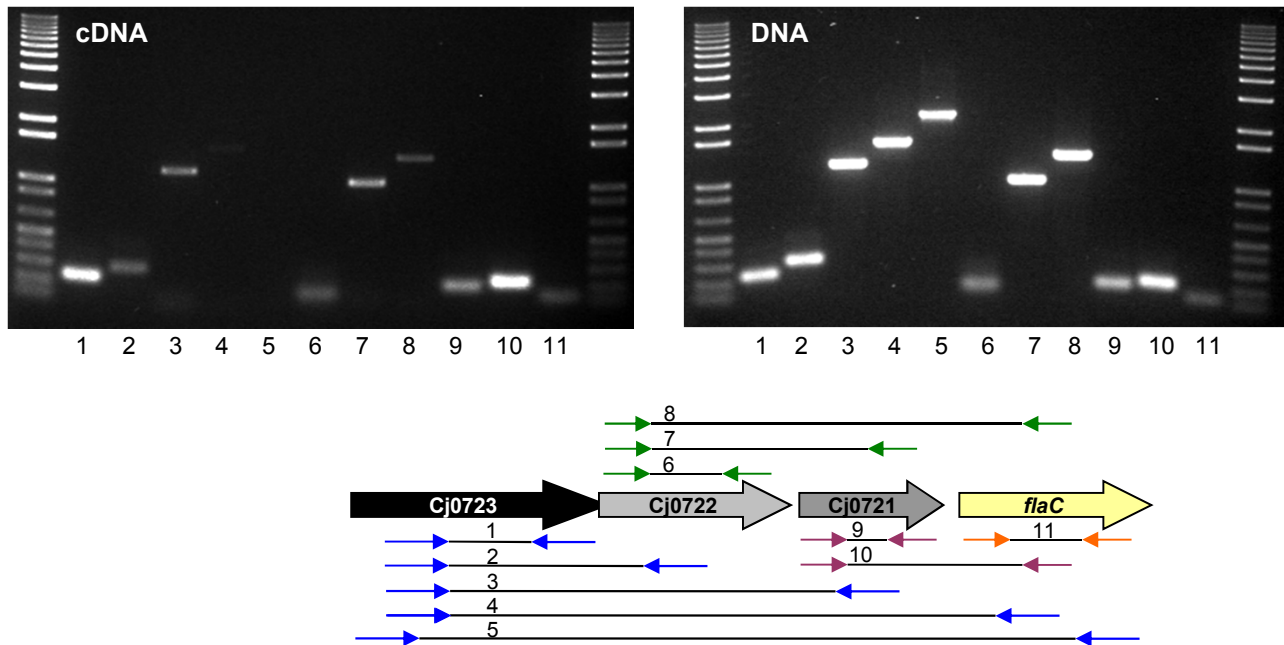

Fig. S3

Supplement: Figure S3 [file sph001160032sf6.pdf]

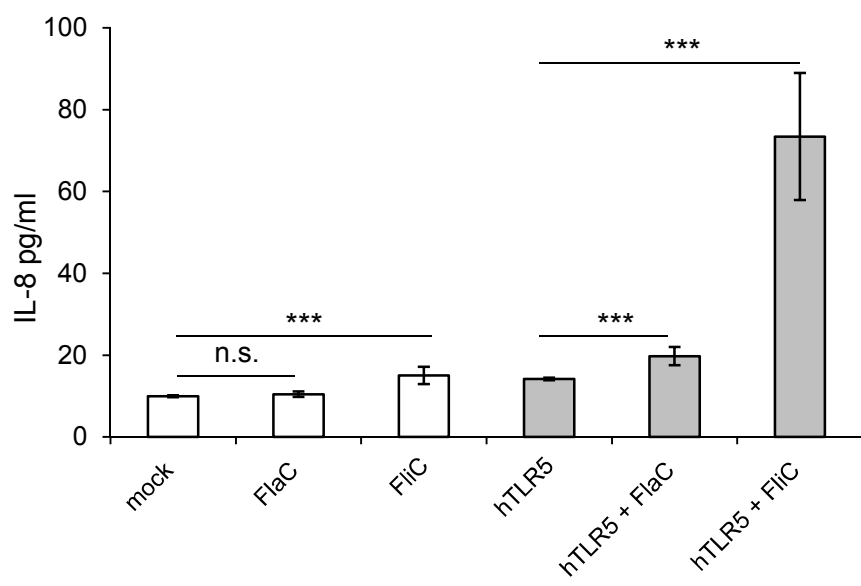

**Fig. S4**

Supplement: Figure S4 [file sph001160032sf7.pdf]

**A**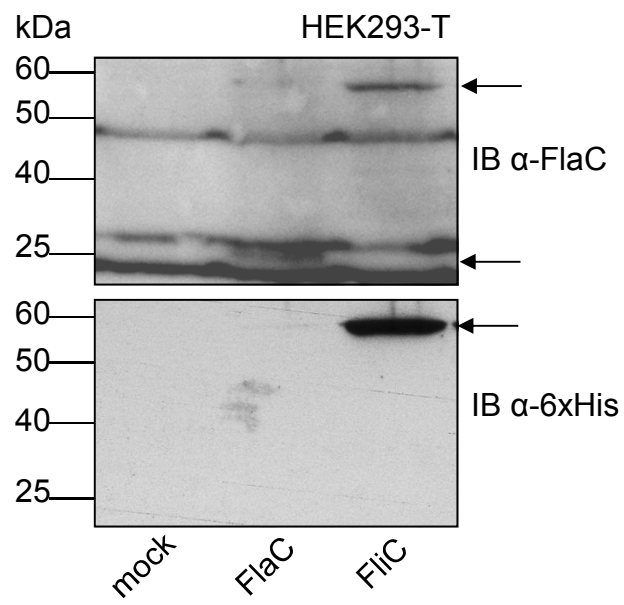**B**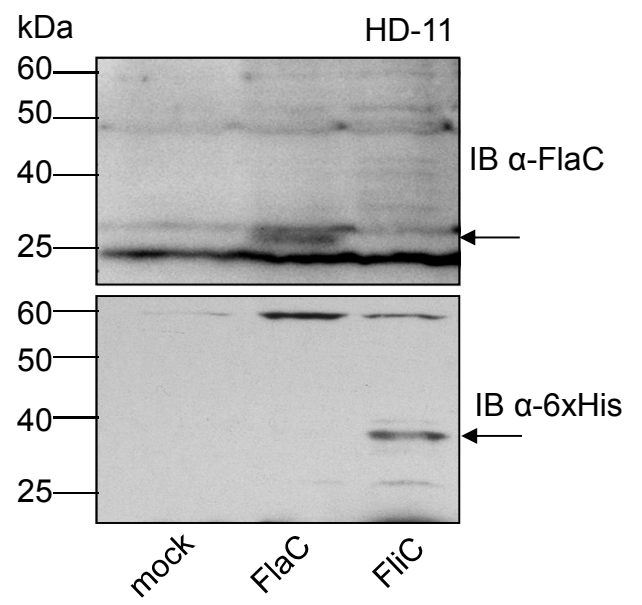

**Fig. S5**

Supplement: Figure S5 [file sph001160032sf8.pdf]

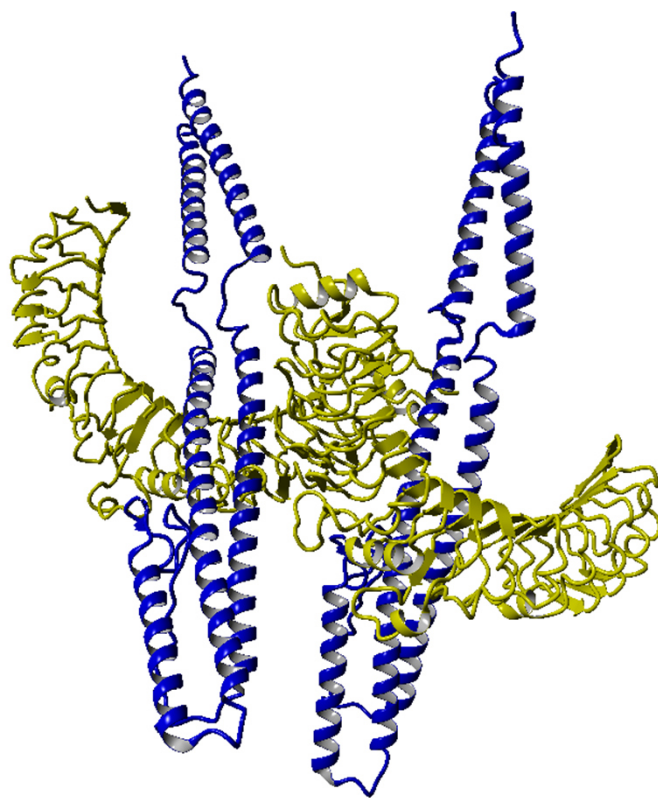

**Fig. S6**

Supplement: Figure S6 [file sph001160032sf9.pdf]

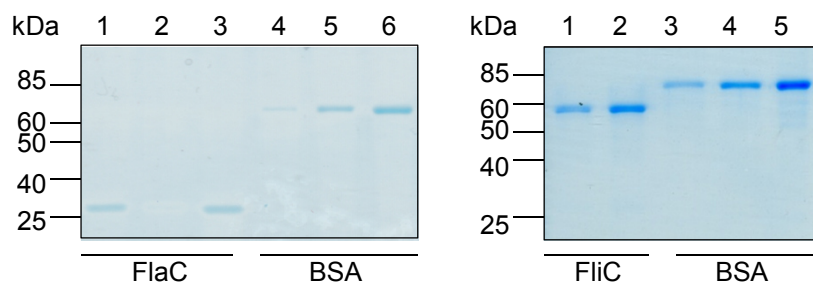

**Fig. S7**

Supplement: Figure S7 [file sph001160032sf10.pdf]
