# Supplementary material for: Novel Immunomodulatory Flagellin-Like Protein FlaC in Campylobacter jejuni and Other Campylobacterales
Source: mSphere. 2015 Dec 2;1(1):e00028-15. doi: 10.1128/mSphere.00028-15 (PMC4863622; doi:10.1128/mSphere.00028-15)
Supplement: Table S1 [file sph001160032st2.pdf]

Supplementary Table S1: Origin of sera from *Campylobacter*-positive and -negative chickens.

Chicken	Feed	Keeping	Age (weeks)	Chicken line	Hygiene status	*Campylobacter* status
1	organic	floor	11	Lohmann Brown	conventional	positive
2	organic	floor	11	Lohmann Brown	conventional	positive
5	organic	floor	11	Lohmann Brown	conventional	positive
6	organic	floor	11	Lohmann Brown	conventional	positive
7	organic	floor	11	Lohmann Brown	conventional	positive
8	organic	floor	15	Lohmann Brown	conventional	positive
9	organic	floor	15	Lohmann Brown	conventional	positive
10	organic	floor	15	Lohmann Brown	conventional	positive
11	organic	floor	15	Lohmann Brown	conventional	positive
12	organic	floor	15	Lohmann Brown	conventional	positive
13	organic	floor	15	Lohmann Brown	conventional	positive
15	conventional	floor	19	Lohmann Brown	conventional	positive
17	conventional	floor	19	Lohmann Brown	conventional	positive
19	conventional	floor	19	Lohmann Brown	conventional	positive
20	conventional	aviary	17	M11	SPF[a]	negative
21	conventional	aviary	17	M11	SPF[a]	negative
22	conventional	aviary	17	M11	SPF[a]	negative

[a]specific pathogen-free
