# Supplementary material for: Novel Immunomodulatory Flagellin-Like Protein FlaC in Campylobacter jejuni and Other Campylobacterales
Source: mSphere. 2015 Dec 2;1(1):e00028-15. doi: 10.1128/mSphere.00028-15 (PMC4863622; doi:10.1128/mSphere.00028-15)
Supplement: Text S1 [file sph001160032s1.pdf]

Supplementary Material

Supplementary Methods

Microbiota analysis of chicken caecum tissue. The microbiota was analysed as described in Yang *et al.* (1), with modifications. Briefly, 16S rRNA gene amplicons were generated from chicken caecal tissue complete DNA preparations by PCR (on 50 ng of DNA) with 454 Lib-L fusion primers containing the target-specific sequences 8F (5'-AGAGTTTGATCCTGGCTCAG-3') and 541R (5'-WTTACCGCGGCTGCTGG-3'), and sequenced from the 3' end using Titanium chemistry on a Roche 454 GS FLX+ instrument. Sequences were pre-processed with the shotgun pipeline of GS Run Processing Software version 3.0 to extract .fna and .qual files. We followed the recommendations for the UPARSE pipeline for additional filtering and quality trimming (http://drive5.com/usearch/manual/uparse_pipeline.html). Sequences were truncated to 300 bp, filtered to a maximum expected error of 1 and clustered into 97% identity Operative Taxonomic Units (OTUs) using UPARSE 7.0.1090 (2). The representative sequences of all resulting OTUs were classified with RDP classifier 2.10.1 (3), retaining classifications with a minimal bootstrap support value of 0.8. OTUs not identified to class level were discarded. No chloroplast or other non-bacterial sequences were identified in the dataset.

For representative sequences identified to genus level with a bootstrap support value of at least 0.97, species level classification was attempted. Sequences were BLASTed against the small ribosomal database of Living Tree Project version 119 (LTPs) (4) and against Ribosomal Database Project release 11.2 (RDP) (5), modified using TaxCollector 2.0 (6) for easier automatic extraction of species information. The species annotation of the top BLAST hits was noted as a possible species identification if the following conditions were met: identity between hit and query sequence 97% or higher, matching region covering at least 97% of the query sequence, no hit sequence with another species annotation producing the same BLAST expectation value, and species annotation matching either the genus identification produced by the RDP classifier or the RDP classifier-based genus annotation of the LTP entry for the type strain of any given species. If the TaxCollector-based and the LTP-based analyses resulted in the same possible species annotations, this species name was added to the classification of the representative sequence. In parallel, in

order to verify and improve the annotation, the majority consensus of the non-subsampled reads was used in a BLAST search against the NCBInr database, which served to assign the reads to a closest bacterial relative. In order to ensure equal sequence numbers during statistical analysis, the dataset was subsampled to 1180 sequences per sample, which corresponded to the final sequence count in the sample with the least sequences.

Further analyses were based on Metastats (7) and mothur (8), which was also used for general sequence handling. R graphics was used to visualize OTU numbers and Shannon index values. A Principal Coordinates Analysis (PC; PCoA) based on Bray-Curtis distances was performed using the R package vegan version 2.0-9 (9).

Supplementary Reference List

1. **Yang I, Eibach D, Kops F, Brenneke B, Woltemate S, Schulze J, Bleich A, Gruber AD, Muthupalani S, Fox JG, Josenhans C, Suerbaum S**. 2013. Intestinal microbiota composition of interleukin-10 deficient C57BL/6J mice and susceptibility to *Helicobacter hepaticus*-induced colitis. PLoS ONE **8**: e70783.

2. **Edgar RC**. 2013. UPARSE: highly accurate OTU sequences from microbial amplicon reads. Nat Methods **10**: 996-998.

3. **Wang Q, Garrity GM, Tiedje JM, Cole JR**. 2007. Naïve Bayesian classifier for rapid assignment of rRNA sequences into the new bacterial taxonomy. Appl Environ Microbiol **73**: 5261-5267.

4. **Yarza P, Richter M, Peplies J, Euzeby J, Amann R, Schleifer KH, Ludwig W, Glöckner FO, Rosselló-Móra R**. 2008. The All-Species Living Tree project: a 16S rRNA-based phylogenetic tree of all sequenced type strains. Syst Appl Microbiol **31**: 241-250.

5. **Cole JR, Wang Q, Fish JA, Chai B, McGarrell DM, Sun Y, Brown CT, Porras-Alfaro A, Kuske CR, Tiedje JM**. 2014. Ribosomal Database Project: data and tools for high throughput rRNA analysis. Nucleic Acids Res **42**: D633-D642.

6. **Giongo A, Davis-Richardson AG, Crabb DB, Triplett EW**. 2010. TaxCollector: Modifying current 16S rRNA databases for the rapid classification at six taxonomic levels. Diversity **2**: 1015-1025.

7. **White JR, Nagarajan N, Pop M**. 2009. Statistical methods for detecting differentially abundant features in clinical metagenomic samples. PLoS Comput Biol **5**: e1000352.

8. **Schloss PD, Westcott SL, Ryabin T, Hall JR, Hartmann M, Hollister EB, Lesniewski RA, Oakley BB, Parks DH, Robinson C, Sahl JW, Stres B, Thallinger GG, Van Horn DJ, Weber CF**. 2009. Introducing mothur: open-source, platform-independent, community-supported software for describing and comparing microbial communities. Appl Environ Microbiol **75**: 7537-7541.

9. **Oksanen J, Blanchet FG, Kindt R, Legendre P, Minchin PR, O'Hara RB, Simpson GL, Solymos P, Stevens MH, Wagner H**. vegan: Community Ecology Package. R package version 2.0-9. 2013.
