# Supplementary material for: Novel Immunomodulatory Flagellin-Like Protein FlaC in Campylobacter jejuni and Other Campylobacterales
Source: mSphere. 2015 Dec 2;1(1):e00028-15. doi: 10.1128/mSphere.00028-15 (PMC4863622; doi:10.1128/mSphere.00028-15)
Supplement: Table S2 [file sph001160032st3.pdf]

Supplementary Table 2: Oligonucleotides used for RT-PCR.

Gene	Primers	Sequence (5'-3')	T_m [a] (°C)	Reference
hGAPDH	hGAPDH_F	GTCGGAGTCAACGGATTTGGTCGT	69	This study
	hGAPDH_R	GACGGTGCCATGGAATTTGCCATG	69	This study
hIL-1β	Hs_IL1B_1_SG		55	Qiagen, QuantiTect
hIL-8	Hs_IL8_1_SG		55	Qiagen, QuantiTect
hIL-10	Hs_IL10_1_SG		55	Qiagen, QuantiTect
chGAPDH	chGAPDH_F2	AGGGTGGTGCTAAGCGTGTT	59	This study (B. K.)
	chGAPDH_R2	AAGGGTGCCAGGCAGTTG	59	This study (B. K.)
chIL-1	chIL-1_F1	CTGAGTCATGCATCGTTTATGTTTC	59	This study (B. K.)
	chIL-1_R1	AAATACCTCCACCCCGACAAG	59	This study (B. K.)
chCXCLi2	chIL-8_F1	CTGGCCCTCCTCCTGGTTTC	59	This study (B. K.)
	chIL-8_R1	TGGCGTCAGCTTCACATCTTG	59	This study (B. K.)
chK203	chK203_RT_F1	CCTGCTGCACCACTTACATAACA	59	This study (B. K.)
	chK203_RT_R1	GCGCTCCTTCTTTGTGATGAA	59	This study (B. K.)
Cj16S rRNA	Cj16S rRNA RT_F	TGCTAGAAGTGGATTAGTGG	53	This study
	Cj16S rRNA RT_R	AAGCCATTACCTTACCAACT	53	This study
CjflaC	CjflaC _RT_F1	AATGCTAAGAACAGAAGCAA	53	This study
	CjflaC _RT_R1	TTCCGCTACCTACTACAAAA	53	This study
